# Elucidating the biochemical basis of *trans*‐16:1 fatty acid change in leaves during cold acclimation in wheat

**DOI:** 10.1002/pei3.10044

**Published:** 2021-05-17

**Authors:** Qiang Li, Wenyun Shen, Ioannis Mavraganis, Liping Wang, Peng Gao, Jie Gao, Dustin Cram, Yifeng Li, Ziying Liu, David Brian Fowler, Youlian Pan, Jitao Zou

**Affiliations:** ^1^ Aquatic and Crop Research and Development National Research Council Canada Saskatoon SK Canada; ^2^ National Key Laboratory of Crop Genetic Improvement Huazhong Agricultural University Wuhan China; ^3^ Department of Plant Sciences University of Saskatchewan Saskatoon SK Canada; ^4^ Digital Technologies National Research Council Canada Ottawa ON Canada; ^5^ Department of Computer Science Brock University Catharines ON Canada

**Keywords:** wheat, phosphatidylglycerol, *trans*‐16:1, cold acclimation

## Abstract

In plant cells, phosphatidylglycerol (PG) in the chloroplast has a characteristic *trans*‐∆3‐hexadecenoic acid (t16:1) at the *sn*‐*2* position. The t16:1 content in wheat leaf tissues decreases during cold treatment, but the significance of this fatty acid compositional change and the underlying biochemical mechanism remains poorly understood. Using a large collection of wheat cultivars displaying a varying capacity of freezing tolerance, we show for the first time under field conditions that this low temperature induced t16:1 change is associated with winter hardiness. To explore the metabolic mechanism responsible for the reduction of t16:1, we performed detailed lipid analysis and comparative transcriptome study with four selected wheat lines under cold acclimation. Our results show that wheat leaf tissues experience a gradual decrease in chloroplast lipid pathway activity during cold acclimation and that the decline in chloroplast lipid synthesis manifests itself in the decrease of t16:1 in PG. Comparative RNA‐seq analyses with leaf tissues further reveal concerted transcriptome shifts indicating a rebalancing of chloroplast and cytosolic lipid synthesis during cold acclimation. Our study, thus, provides mechanistic understanding on chloroplast lipid adjustments as a “molecular ideotype” and the t16:1 change as a specific metabolite marker for screening freezing tolerance in wheat.

## 1 INTRODUCTION

Cold acclimation in crops requires a period of exposure to low temperatures before reaching fully hardened state, and this process is dependent on both how low the temperatures the plants are exposed to and the length of the low temperature that the plants have experienced (Limin & Fowler, 2002). Development of acclimation involves alterations in gene expression profiles, metabolic adjustment, and physiological changes (Li et al., 2018; Gordon‐Kamm & Steponkus, 1984; Guy, 1990). Metabolic shifts concerning membrane lipid metabolism are integral parts of this process. Studies using protoplast systems showed that cold acclimation alters dramatically the properties of plasma membrane during freeze‐induced osmotic contraction, and as a result plant cells suffer less damage under severe freezing conditions (Gordon‐Kamm & Steponkus, 1984). It has been reported in cereal crops that low temperature induces decrease in *trans*‐∆3‐hexadecenoic acid (t16:1) in the fatty acid profiles of leaf tissues and that there was a correlation between the decrease in t16:1 and freezing tolerance when examined under controlled environments (Huner et al., 1989). A more recent study showed that the production of t16:1 is linked to the redox status of the chloroplast (Horn et al., 2020). However, the physiological relevance of t16:1 to freezing tolerance, if any, is unclear (Bishop, 1986; Gray et al., 2005).

In the plant cells, the only glycerolipid molecule species containing t16:1 is the chloroplast phosphatidylglycerol (PG). In the chloroplast, PG synthesis starts from glycerol‐3‐phosphate (G‐3‐P), which is acylated sequentially at the *sn*‐*1* and *sn*‐*2* positions through two acyltransferases, glycerol‐3‐phosphate acyltransferase (GPAT), and *lyso*‐phosphatidic acid acyltransferase (LPAAT), leading to the formation of phosphatidic acid (PA). Due to substrate preference of the chloroplast LPAAT, PA produced within the chloroplast has a 16:0 fatty acid incorporated at the *sn*‐*2* position of the glycerol backbone, whereas in the *sn*‐*1*, 18 carbon fatty acids (18:0 or 18:1) are preferred. PA is converted to CDP‐diacylglycerol (CDP‐DAG) by CDP‐DAG synthase (CDS), which is then converted to phosphatidylglycerolphosphate (PGP) by phosphatidylglycerol phosphate synthase (PGPS). Subsequent dephosphorylation of PGP by phosphatidylglycerolphosphate phosphatase (PGPP) produces PG. The *sn*‐*2* 16:0 in PG is then desaturated by fatty acid desaturase 4 (FAD4), which is highly substrate specific, and generates the unusual t16:1 in PG (Gao et al., 2009). Desaturation of the *sn*‐*1* C18‐fatty acid in PG can be proceeded through FAD6, FAD7, and FAD8, resulting in the polyunsatured 18:3. Consequently, the t16:1 fatty acid is primarily found in PG (34:4) that has a fatty acid distribution of 18:3 at *sn*‐*1* and t16:1 at *sn*‐*2* (Browse et al., 1986; Ohlrogge & Browse, 1995; Schmidt & Heinz, 1993). In cereals, major chloroplast lipids such as monogalactosyl diacylglycerol (MGDG) and digalactosyl diacylglycerol (DGDG) are originated from the eukaryotic pathway in the endoplasmic reticulum (ER) systems of the cytosolic compartment. PG is the only class of lipids that depends exclusively on the chloroplast lipid pathway.

There are strong interests in developing techniques to assess the capacity of freezing tolerance through quantifiable physiological, metabolic, or molecular parameters. These parameters, be it the level change of a particular metabolite, expression profile of a specific gene, or unique expression of an exceptional gene allele, can be useful “molecular ideotypes” indicative of crop tolerance to stress conditions. Defining molecular ideotype is also important for dissecting molecular mechanisms underpinning complex traits such as freezing tolerance. In this study, we investigated the dynamics of t16:1 during cold acclimation in wheat. Our results reveal the biochemical mechanism of t16:1 decrease during cold acclimation and provides support for future studies on how the regulation of the chloroplast lipid pathway is related to the development of winter hardiness in wheat.

## 2 MATERIALS AND METHODS

### 2.1 Plant materials and growth conditions

Wheat cultivars used in field trials of this study (Table 1) have been described by Byrns et al. (2020). The procedure outlined by Fowler (2008) was used to determine the LT50 (lethal temperatures at which 50% of a population is killed in an artificial freeze test) of each genotype at the end of each cold acclimation period. Briefly, crowns of 35 cultivars at three leaf‐stage were covered with moistened sand in an aluminum weighing can, placed in a programmable freezer and held at −3°C for 12 h. Once the freezer is stabilized, it was programmed by 2°C decrease per hour till −17°C, and thereafter reduce the temperature by 8°C per hour. Six to ten crowns were removed at 2°C intervals each genotype in each treatment. Samples were then thawed overnight at 4°C and subsequently transplanted into soil plate for regrowth at 20°C for 21 days. Plant recovery was counted at each temperature (survivor vs. dead) and LT50 was calculated for each treatment. Each test was replicated three times.

**TABLE 1:** LT50 values of the wheat cultivars tested in the field trials. LT50 denotes a temperature at which 50% of a population would be perished under artificial freezing test

No.	Cultivar	LT50(°C)	No.	Cultivar	LT50(°C)	No.	Cultivar	LT50(°C)
1	Manitou	−8.3	13	Gaines	−16.67	25	Lancota	−19.56
2	Winter Manitou	−13.2	14	Sprague	−16.89	26	Abilene	−19.77
3	Spring Norstar	−13	15	McClintock (UM508)	−17	27	Sundance	−19.78
4	CappelleDesprez	−14	16	Karl 92	−17.44	28	Froid	−20
5	Dawnson's Golden	−15	17	Jagger	−17.56	29	Winalta	−20.22
6	Genessee	−15.11	18	AC Tempest	−17.89	30	Minter	−20.44
7	Jonas‐Fife	−15.11	19	Mironovskaja 808	−18.89	31	Norwin	−20.51
8	Yorkstar	−15.55	20	CDC Falcon	−19	32	Ulianovka	−20.89
9	TAM 200	−15.78	21	Redwin	−19.11	33	CDC Clair	−21.01
10	Besostaja	−16.11	22	Yogo	−19.22	34	Alabaska	−21.39
11	Blueboy	−16.27	23	Cheyenne	−19.33	35	Norstar	−21.7
12	Frederick	−16.67	24	Roughrider	−19.44			

For cold treatment under controlled environments, four wheat cultivars (Manitou [MA], Norstar [NO], winter Manitou (WM), and spring Norstar [SM]) were grown in chambers with 16‐h‐light (~120 μmol m^−2^ s^−1^) and 8‐h‐dark at 23°C up to the stage of four leaves (3 weeks) and then transferred to different temperatures for cold treatment. The third fully opened leaves from untreated and cold‐treated plants was collected and immediately frozen in liquid N_2_. Samples were stored at −80°C until lipid analysis. Field testing experiments were performed at the University of Saskatchewan Kernen research farm in Saskatoon, Saskatchewan, Canada. Wheat cultivars were seeded in the field on August 29 in 2013 and 2014 in two replicates for each year. Leaf samples were collected on September 23 (13.4°C) and October 23 (3.5°C) in the 2013 trial and September 22 (13.7°C) and October 30 (2.2°C) in the 2014 trial. The percent decrease in t16:1 after cold acclimation in October was calculated relative to the t16:1 content in leaves collected in September. The 2014 samples were left for 5 weeks of cold treatments due to the occurrence of abnormal high‐temperature weather fluctuations. All leaf samples were frozen with liquid N_2_ immediately after harvesting and store at −80°C.

### 2.2 Lipids and fatty acids analysis

Total lipid extraction was performed according to Shen et al. (2010). Briefly, 0.5 g of plant material was ground into powder under liquid N_2_ with a mortar and pestle. The powder was further dissolved in 4 ml chloroform/methanol/formic acid (10:10:1, v/v/v) and transferred into a glass tube with a Teflon‐lined screw cap. Following centrifugation at 2,000 g for 2 min, the upper phase was transferred into a new tube, and the tissue pellet was re‐extracted with 2 ml of chloroform/methanol/water (5:5:1, v/v/v). The two extractions were combined and washed with 3 ml of 0.2 M H_3_PO_4_. After centrifugation at 2,000 g for 2 min the lower chloroform phase was removed into a new tube and dried under N_2_. After dissolving in 0.2 ml of chloroform, total lipids were separated by two‐dimensional thin layer chromatography (2D‐TLC) or one‐dimensional TLC (1D‐TLC). In the controlled‐condition experiments, PG was separated by 2D‐TLC on Silica Gel 60 (EMD Chemical, Germany) using chloroform/methanol/50% ammonia hydroxide (65:35:2, v/v/v) for one direction and chloroform/methanol/acetic acid/water (85:15:10:3, v/v/v/v) for the second direction as was previously described by Li et al. (2015). For the field trial lipid analysis, PG was isolated by 1D‐TLC using a modified mobile phase of acetone/toluene/water (70:30:6, v/v/v). The developed TLC plates were then sprayed with primulin staining solution (0.05% primulin in 80% acetone), and the lipid classes were visualized under UV light. The PG fraction was scraped from the TLC plate for fatty acid composition analysis as described previously (Shen et al., 2010). Briefly, the PG fraction was transmethylated with 3 N methanolic/HCl at 80°C for 2 h. The fatty acid methyl esters (FAMEs) were then extracted twice using 2 ml of hexane and dried under nitrogen gas. FAME samples were resuspended in 20–50 μl of hexane and analyzed in a Hewlett‐Packard 5890A gas chromatograph (GC) equipped with a DB‐23 column (30‐m × 0.25‐mm) with a 0.25‐μm film thickness and an FID detector. The column temperature was maintained at 160°C for 1 min, and then raised to 240°C at a rate of 4°C/min.

### 2.3 Stereo‐specific analysis

Fatty acid composition at the *sn*‐*2* position of PG was determined as previously described (Shen et al., 2010). Total lipids were separated by 2‐D TLC, and the PG fraction was eluted from the silica plate by methanol/chloroform, 2:1. After drying under N_2_, PG was dissolved in 1 mL 40 mM Tris‐HCl buffer, pH 7.2, and 0.05% Triton X‐100 containing 50 mM H_3_BO_3_. PG digestion was initiated by the addition of 3000 units of *Rhizopus arrhizus* lipase, and the mixture was incubated at 37°C for 1 h. The lipid digestion reaction was stopped by the addition of 1 ml of 0.15 M acetic acid. Total lipids were then extracted with 3.8 ml of chloroform/methanol (2:1, v/v) solution through centrifugation, and the organic phase was then loaded in silica G20 plates. Lipid classes were separated through TLC in chloroform/methanol/acetic acid/water (85:15:19:3, v/v/v/v) as the mobile phase. After visualization with primulin staining solution the *lyso*‐lipid fraction containing fatty acids at the *sn*‐2 position was collected and prepared for gas chromatography. Fatty acid composition at the *sn*‐1 was calculated based on the fatty acid compositions of the original diacyl glycerolipid and the *lyso*‐lipid.

### 2.4 Lipidomic analysis

Lipid extraction for lipidomics analysis was performed as described by Welti et al. (2002). Leaf and crown tissues were heated at 75°C in 2 ml isopropanol supplemented with 0.01% butylated hydroxytoluene. Lipids were extracted with chloroform/methanol (2:1, v/v) several times until the leaf or crown tissues became colorless. The combined extracted solutions were then washed with 1 M KCl. After centrifugation, the chloroform phase was removed and dried under nitrogen gas. Lipidomics analysis was performed by ESI‐MS/MS at the Kansas Lipidomic Research Center.

### 2.5 Putative FAD4 cloning and real‐time PCR

Total RNA was extracted from leaf tissues using the Plant Mini kit (Qiagen) according to manufacturer's instructions, and its quantity and quality were measured with the NanoDrop 2000. One μg of total RNA was used for cDNA synthesis using the QuantiTect Reverse Transcription kit (Qiagen). Specific primers for three short sequences identified from the MA transcript database with high homology to *Brachypodium* FAD4 were used for PCR amplification. The 5’ and 3’ ends of the putative FAD4 genes were obtained using the SMARTer RACE 5’/3’ kit (Clontech). The derived fragments were then cloned into pGEM‐T (Promega) and verified by sequencing, resulting in a single cDNA that encodes for 283 amino acids. RT‐PCR was performed to monitor the expression of the putative wheat *FAD4* gene with specific primers. Wheat *actin* (GenBank: AB181991) was used as reference gene for RT‐PCR. The specificity of all primers were checked with BLASTn searches. Primers used in this experiment are listed in Table S5.

### 2.6 RNA sequencing and data analysis

Total RNA was extracted from 0.1 g wheat leaf tissues for each of the non‐treated and treated samples using the Agilent Plant RNA isolation kit (Agilent Technologies). A total of 24 cDNA libraries (three biological replicates for each sample) were prepared according to Illumina's instructions, using the TruSeqRNA Sample Preparation Kit v2 (Illumina). Pair‐end sequencing (126 cycles) was conducted on Illumina HiSeq 2500 following the manufacturer's protocols. We trimmed adaptor sequences, discarded low‐quality reads (Phred Score <=20, http://www.phrap.com/phred), and eliminated short reads (length <= 20 bps) using a software package from Stanford (De Wit et al., 2012). STAR (v2.4.2a, Dobin et al., 2013) was used for mapping reads and generating raw count/gene/sample. IWGSC Version 2.2 survey genome assembly was used as reference (IWGSC, 2014). Unmapped reads were submitted to Trinity (v2.1.1, Haas et al., 2013) for de novo assembly with default parameters. A total of 157,566 genes were identified including 99,386 from reference‐guided assembly and 58,180 from de novo assembly in the data sets.

DESeq2 (Love et al., 2014) was applied for data normalization across all 24 samples and subsequent DEG identification (|log2FC| ≥ 1, *p* ≤ .01 and >50 read counts across all samples) for each treatment–control pair (MA‐CvsMA, WM‐CvsWM, NO‐CvsNO, and SN‐CvsSN, where, WM = winter Manitou, MA = Manitou, NO = Norstar, SN = spring Norstar, C = cold).

Clustering and correlation analysis were performed as described in Pan et al. (2018). Known orthologs in *Arabidopsis thaliana*, *Brachypodium distachyon*, *Oryza sativa* (ssp. *japonica*), and *Zea mays* among the 99,386 IWGSC were sought from EnsemblePlants v28. The orthologs in *A*. *thaliana* among 58,180 genes from the de novo assembly were obtained through BlastX against protein database from TAIR10 (https://www.arabidopsis.org/) with the threshold criteria of *p* ≤ 10–6, minimum alignment (peptide) length ≥50, Similarity ≥50%, and bit score ≥50. GOAL software (Tchagang et al., 2010) was used in the gene ontology (GO) enrichment analysis. RNA sequencing raw and analyzed data have been submitted to the Gene Expression Omnibus (GEO).

## 3 RESULTS

### 3.1 Reduction of t16:1 during cold acclimation under field conditions

**FIGURE 1:**
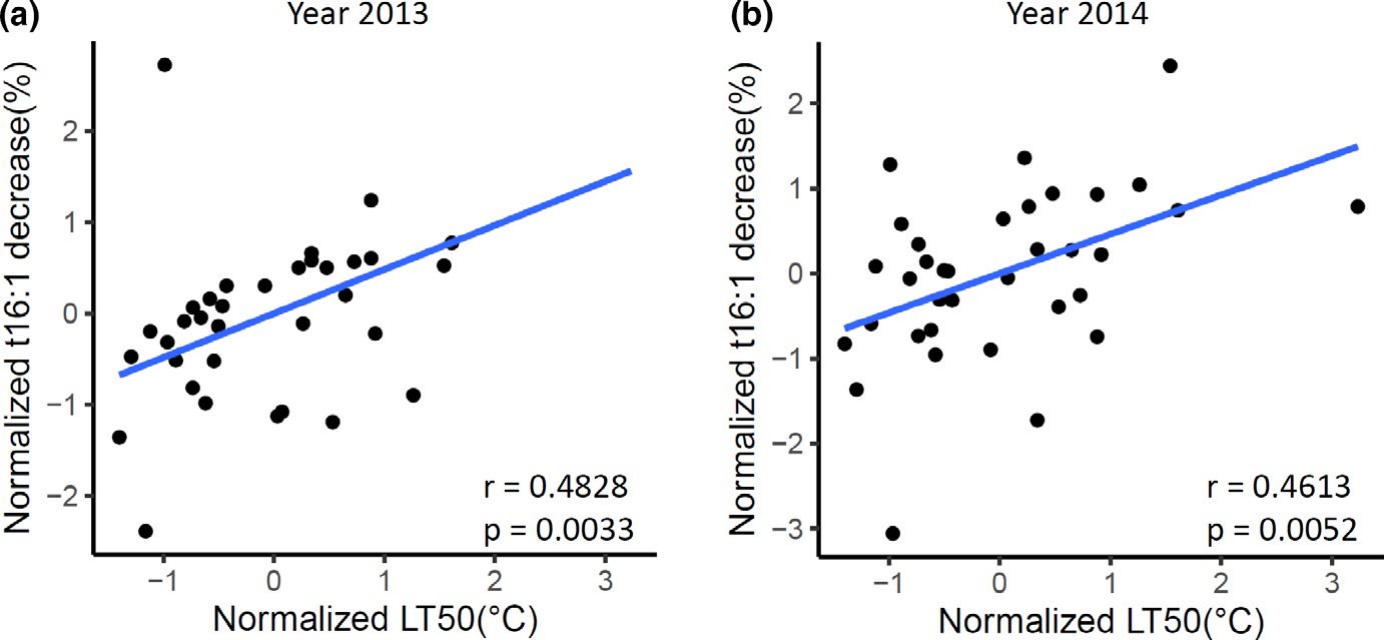
Relationship between LT50 and decreases of t16:1 in phosphatidylglycerol (PG) after cold acclimation in the field. Wheat cultivars were grown in the field close to Saskatoon, Saskatchewan, Canada, and leaf tissue harvesting began in late September (soil temperature at 14°C, control). The levels of t16:1 in PG were monitored 4 or 5 weeks later (soil temperature at 2°C, cold‐acclimated). The percentage decrease of t16:1 after cold acclimation was calculated relative to the t16:1 content in the controls. Field trial data from 2013 (a) and 2014 (b) were presented. Values presented are means of three replicates. The LT50 values of these lines have been determined as described in Fowler (2008). LT50 values and the percentage decreases of t16:1 after cold acclimation were subjected to *z*‐transformation before calculating Pearson correlation coefficient to assess their relationships

The prospect of t16:1 as a prognostic marker of freezing tolerance required experimental validation under field conditions. We conducted field trial in two consecutive years using a collection of 35 wheat cultivars that have LT50 ranging from −8.3 to −25°C (Table 1). The trial population was sown at the end of August. Samples were harvested on September 23 (13.4°C, control) and October 23(3.5°C, cold‐treated) in the 2013 trial, and September 22 (13.7°C, control) and October 30 (2.2°C, cold‐treated) in the 2014 trial, due to field temperature variations between the two years (Figure S1). Total lipids were extracted from leaf tissues, and fatty acid methyl‐esters were prepared for fatty acid profile analysis. Mol % of each fatty acid were calculated as previously described (Li et al., 2015). The percentage decrease in t16:1 observed after cold (samples collected in October) was calculated relative to the t16:1 content in the control (samples collected in September). After plotting the percentage decrease in t16:1 with LT50 values for the 35 cultivars, a positive correlation (Pearson correlation coefficient, *r* = 0.4828, *p* = 0.0033 in year 2013, and 0.4613, *p* = 0.0052 in year 2014) was observed in the field trial in both year 2013 and 2014 (Figure 1). These results extend findings from previous studies with plants raised under controlled environment (Huner et al., 1989) and confirmed that the reduction of t16:1 in leaves of cereal crops is correlated with development of cold tolerance in field conditions.

### 3.2 Reduction of t16:1 after cold acclimation in two pairs of wheat NILs differing in vernalization alleles

Although many genetic factors could affect cellular lipid composition, we next focused on a set of reciprocal near‐isogenic lines (NILs), the winter habit NO, its NIL SN, spring habit MA, and its NIL WM (Laudencia‐Chingcuanco et al., 2011; Li et al., 2018). The SN was generated from a winter genotype NO but harboring the *VRN*‐*A1* gene, whereas the WM was near isogenic to MA except the *vrn*‐*A1* allele (Limin & Fowler, 2002). The LT50 values of NO, SN, WM, and MA were previously determined as −21.7, −13, −13.3, and −8.3°C, respectively (Li et al., 2018).

We grew the four cultivars at 22°C for 3 weeks and then transfer to 18, 15, 10, 8, and 4°C for cold treatment, separately, for 6 weeks. Major glycerolipids were separated through 2D TLC, and the PG spots were scraped off from TLC plates, followed by gas chromatography analysis of fatty acid compositions. The proportion of t16:1 in PG was decreased after 6 weeks of cold treatment (Figure S2). NO, the most winter hardy wheat cultivar, exhibited a 56.8% reduction in t16:1, whereas the spring wheat MA displayed a decrease of 28.6% only. WM and SN, which share similar LT50, experienced a comparable decrease, at 38% and 37% at 4°C, respectively, despite harboring contrasting vernalization (*VRN*‐*A1*) loci, suggesting that the vernalization loci had no direct influence on t16:1 changes.

In an attempt to examine the effect of cold exposure length on t16:1, the four wheat cultivars were grown at 4°C for 2, 4, and 6 weeks under low temperature (Figure S3). MA, WM, and SN experienced smaller changes in t16:1 content between the 4‐ and 6‐week treatments, but the levels in NO continued to decrease. We also analyzed PG from crown tissues harvested from plants grown for 6 weeks at 4°C. Our results show that t16:1 in PG was barely detectable in the crown tissue samples (Figure S4). Hence, as expected from cells of nonphotosynthetic tissues, t16:1 is not present at a significant level in crown tissues.

### 3.3 Fatty acid desaturase 4 was not a causal factor to the reduction of *trans*‐16:1 level

Given that the desaturation of 16:0 to t16:1 in PG is mediated by fatty acyl desaturase FAD4 (Gao et al., 2009), we considered the possibility of a decreased FAD4 causing lower t16:1. We identified three short sequences of 200–300 bp from the MA transcript database previously reported by Li et al. (2018), which exhibit high homology to the *Arabidopsis* FAD4 and *Brachypodium* FAD4 (Figure S5). We performed 5’‐RACE and cloned one wheat FAD4 cDNA homolog, which encodes for a polypetide of 283 amino acids and contains the conserved domains identified previously in the *Arabidopsis* FAD4 (Figure S6). We designed gene‐specific primers for semiquantitative RT‐PCR to monitor the expression profile of this wheat putatitve FAD4 gene. Our results showed that the expression level of the putative *FAD4* in wheat was substantially upregulated during cold acclimation (Figure S7). Although we cannot rule out the possibility that post‐transcriptional regulation was involved in FAD4 activity control, our data suggest that the decrease in t16:1 was unlikely caused by a reduced expression of the FAD4 gene.

### 3.4 Leaf tissues had reduced quantity of chloroplast membrane lipids and increased proportion of ER membrane phospholipids after cold acclimation

Phosphotidylglycerol is the only class of phospholipid in chloroplast, representing about 10% of glycerolipids (Shen et al., 2010). Other phospholipids are present primarily in the ER membrane systems of the cytosolic compartment. We next examined quantitative changes in all glycerolipids after 6 weeks of cold acclimation (Table S1–S4). Low temperature caused an increase in the level of DGDG but a large decrease in MGDG in all four tested lines. Due to the fact that MGDG is the most prominent chloroplast membrane lipid species, the net change combining MGDG and DGDG was an overall reduction in the content of lipids making up chloroplast membranes. Consistent with this, our lipid analysis results showed increases in the proportions of phospholipids that are structural elements for the ER and other membrane systems in the cytosolic compartment, particularly phosphatidylcholine (PC). These results indicated a proportional decrease in the glycerolipid components building up chloroplast membranes after cold acclimation, contrasted by increases in the cytosolic ER membrane systems.

### 3.5 Stereo‐specific analysis of phosphatidylglycerol indicated a rebalanced glycerolipid pathways in leaves during cold acclimation

**FIGURE 2:**
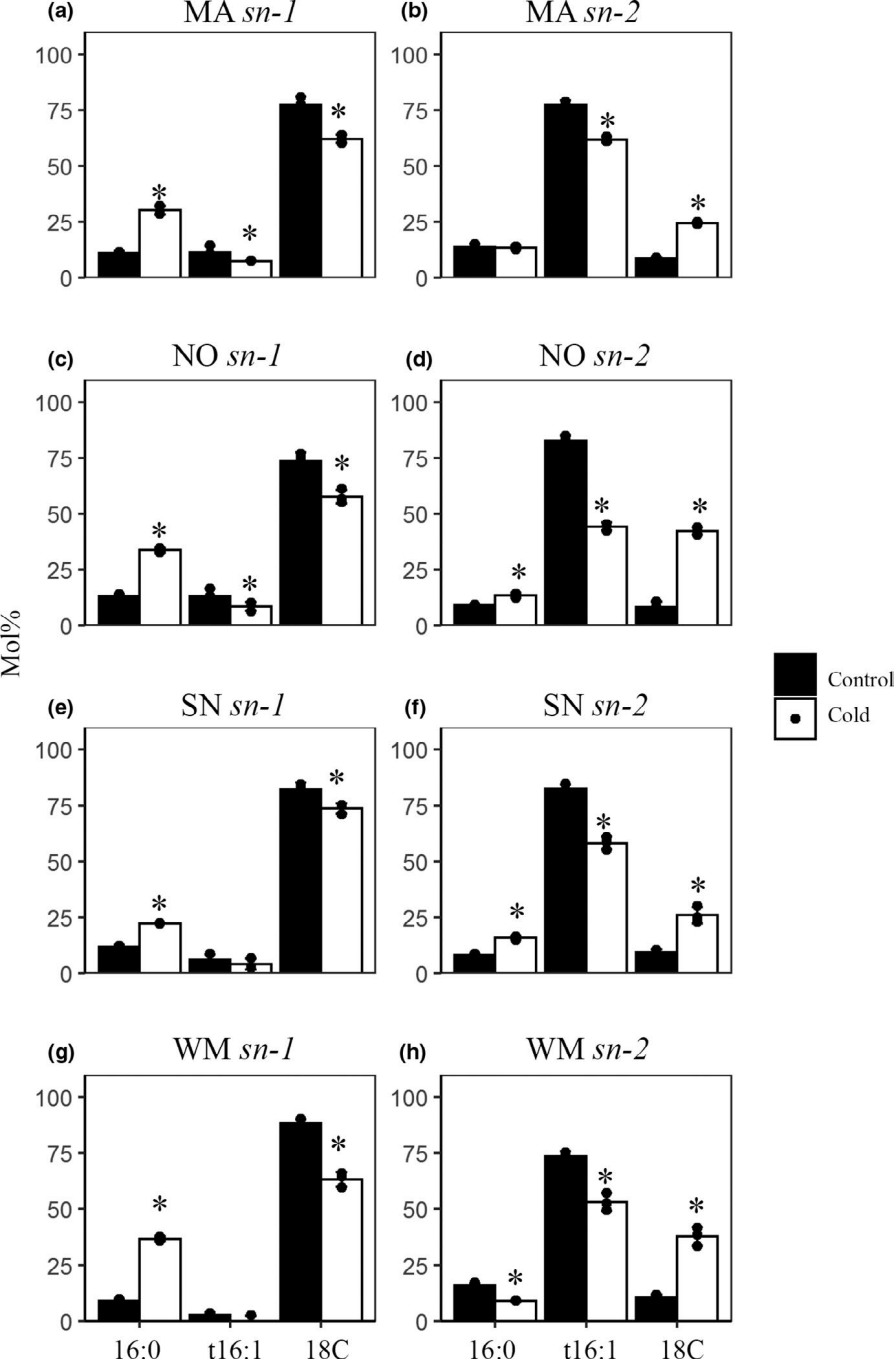
Stereo‐specific analysis of fatty acid compositions in phosphatidylglycerol (PG) from cold‐acclimated (4°C) and non–cold‐acclimated (22°C) leaves. Fatty acid composition at the *sn*‐1 position (a, c, e, and g) and the *sn*‐2 position (b, d, f and h) of PG are shown. 18C represents the sum of 18:0, 18:1, 18:2, and 18:3 fatty acids. MA, Manitou; WM, winter Manitou; NO, Norstar; SN, spring Norstar. Values are expressed as molar percent of fatty acids in PG. Statistically significant differences (two‐tailed student's *t* test) were calculated from three biological replicates (*n* = 3) between cold treatment (4°C) to control (22°C), respectively. *p < .05

The majority of PG is synthesized through the prokaryotic pathway within the chloroplast, but there was also a small portion originating from the cytosolic glycerolipid pathway (Gardiner & Roughan, 1983; Schmidt & Heinz, 1993). The relative contributions of the two pathways to the total cellular PG can be assessed based on fatty acid composition of PG at the *sn*‐*2* position, with the molar percent of C16 fatty acids being indicative of the relative input from the chloroplast pathway. We treated PG with *Rhizopus* lipase to assess the fatty acid composition at the *sn*‐*2*. The derived *lyso*‐PG was further analyzed for its fatty acid composition in *sn*‐1. As shown in Figure 2, t16:1 was found to account up to 80% of the total fatty acids at the *sn*‐2 of PG and its presence at the *sn*‐*1* was at ~10% only. Following cold acclimation, there was a decrease in t16:1 at the *sn*‐2, accompanied by increases of C18 fatty acids. These results strongly suggested that there was a reduced activity from the chloroplast lipid pathway and increased contribution from the cytosolic pathway to PG synthesis in leaf tissues during cold acclimation.

### 3.6 Leaf and crown tissues experienced distinctive lipid profile changes during cold acclimation

**FIGURE 3:**
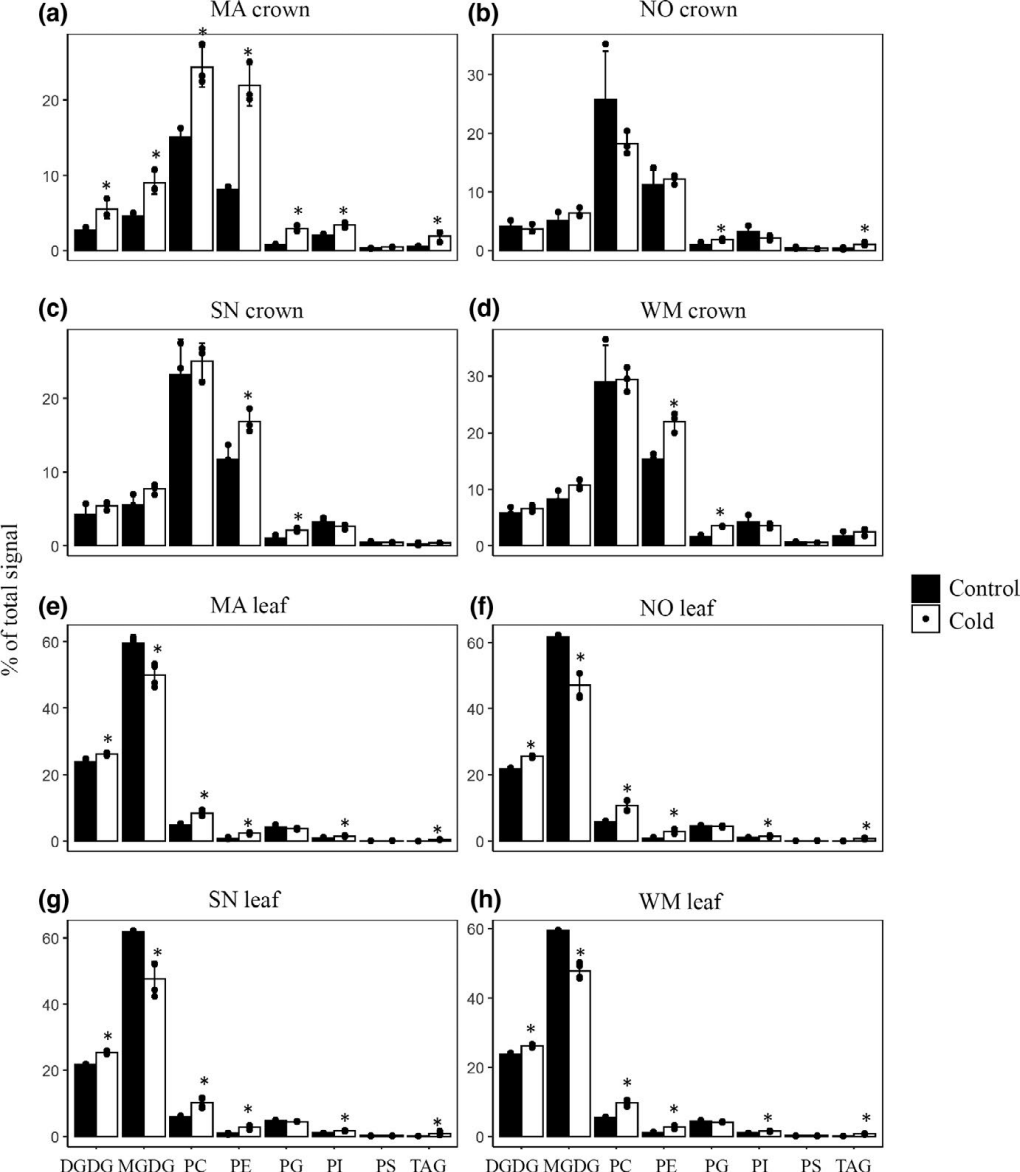
ESI‐MS/MS analysis of glycerolipid species in leaf (a–d) and crown tissues (e–h) before and after cold acclimation. Composition of major lipid classes (%) in the leaves and crowns of four wheat lines grown at 22°C (black bar) and 4°C (white bar) were shown. Detailed molecular composition of all lipid species are available in the Data S1. MA, Manitou; WM, winter Manitou; NO, Norstar; SN, Spring Norstar. DGDG, digalactosyldiacylglycerol; MGDG, monogalactosyldiacylglycerol; PC, phosphatidylcholine; PE, phosphatidylethanolamine; PG, phosphatidylglycerol; PI, phosphatidylinositol; PS, phosphatidylserine; TAG, triacylglycerol. Statistically significant differences (two‐tailed Student's *t* test) were calculated from four biological replicates (*n* = 4) between cold treatment (4°C) to control (22°C), respectively. *p < .05

We next performed lipidomic analysis, which yielded information on all glycerolipid molecules to the level of the head group, carbon chain length, and degree of unsaturation of the acyl groups (Welti et al., 2002). In crown tissues, as expected from a nonphotosynthetic cells, MGDG and DGDG are very low, and PC and phosphatidylethanolamine (PE) are the predominant glycerolipid species. After cold acclimation (Figure 3), leaf tissues experienced decreases in the proportion of MGDG, but increases in DGDG, PC, and PE. The overall level of PG remained relatively unchanged. In crowns, the proportions of MGDG and DGDG were slightly increased, and large increases in PE and PG were observed. It is also noteworthy that both leaf and crowns had increases in the level of triacylglycerol (TAG) during cold acclimation (Figure 3).

Fatty acid composition changes were detected in most of the glycerolipid species after cold acclimation. In keeping with previous observations that cellular lipid fatty acid desaturation index increases under cold temperatures (Falcone et al., 2004), glycerolipids containing polyunsaturated fatty acids including MGDG (36:6), DGDG (36:6), PC (36:6), PC (36:5), PE(36:6), and PE(36:5) were increased while less desaturated lipid such as MGDG (36:5), DGDG(34:3), PC(34:2), PC(34:1), PE(36:4), and PE(34:2) were decreased (Data S1). Because we were specifically interested in the dynamics of t16:1, we looked into PG34:4 (18:3/16:1), which contained 18:3 at the *sn*‐1 and t16:1 at the *sn*‐2. As shown in Figure S8, PG 34:4 in leaf tissues experienced a significant decrease after cold acclimation, with a trend that largely followed LT50. These results further supported the notion that the chloroplast lipid pathway activity was reduced during cold acclimation. In crown tissues, PG34:4 was barely detectable irrespective of before or after cold acclimation, consistent with analysis of TLC‐purified PG where we could not find meaningful level of t16:1 in crown tissues.

### 3.7 Transcriptional regulations in lipid pathways during cold acclimation

Transcriptome analyses of the four wheat lines have been reported using both microarray (Laudencia‐Chingcuanco et al., 2011) and RNA sequencing (RNA‐seq; Li et al., 2018). But previous efforts were focused exclusively on crown tissues. To further investigate the underlying biochemical mechanisms of t16:1 decrease during cold acclimation, we performed RNA‐seq analysis on leaf tissues of the four wheat varieties. On average, 34 million reads per sample were captured. For each sample, the raw RNA‐seq sequence reads are mapped to 99,386 known wheat genes in the IWGSC 2.2 survey genome assembly. On average, 88% of the reads were successfully aligned (See Data S2 for detailed results). The remaining 12% unmapped reads from the initial alignment were further mapped to the *de novo* assembled contigs (see Section 2 for description of de novo assembly procedure). Our results showed that 80% of these unmapped reads were successfully aligned to 58,180 contigs in de novo assembly. Collectively, more than 96% of these reads were mapped. Further applying the filtering criteria (|log2 fold‐change|≥1, *p*‐value ≤ .01 and the maximum read counts in a pair of compared samples ≥50, detailed in the Section 2) on the normalized data and differential expression analysis, we obtained 29,491 differentially expressed genes (DEGs; Data S3).

To investigate the dynamic transcriptional changes in the four wheat varieties after cold acclimation, we performed hierarchical clustering analysis with DEGs. A total of 28 clusters were identified with distinct expression patterns (Figure S9). The resulted cluster membership of each gene is available in Data S3. Each cluster revealed specific expression pattern of genes. For example, genes in DEG6 and DEG9 were consistently downregulated, whereas genes in cluster DGE23 were consistently upregulated after cold treatment in all four wheat lines. On the other hand, some clusters showed specific gene expression patterns in one or two cultivars. For instance, genes in cluster DEG11 were highly induced by cold only in NO and mainly enriched in biological processes such as proteasome core complex assembly, response to misfolded protein, translation, and photorespiration (Data S4 and Figure S10). Genes in DEG23 were upregulated by cold in all four cultivars and highly enriched with protein phosphorylation, transmembrane transport, and metabolic process. In contrast, genes in cluster DEG9 were downregulated by cold and highly enriched in biological processes such as thylakoid membrane organization, carotenoid biosynthetic process, and rRNA processing. Overall, these results were consistent with the supposition that chloroplast pathways were repressed during cold acclimation (Javadian et al., 2010).

### 3.8 Lipid metabolic network adjustments during cold acclimation

**FIGURE 4:**
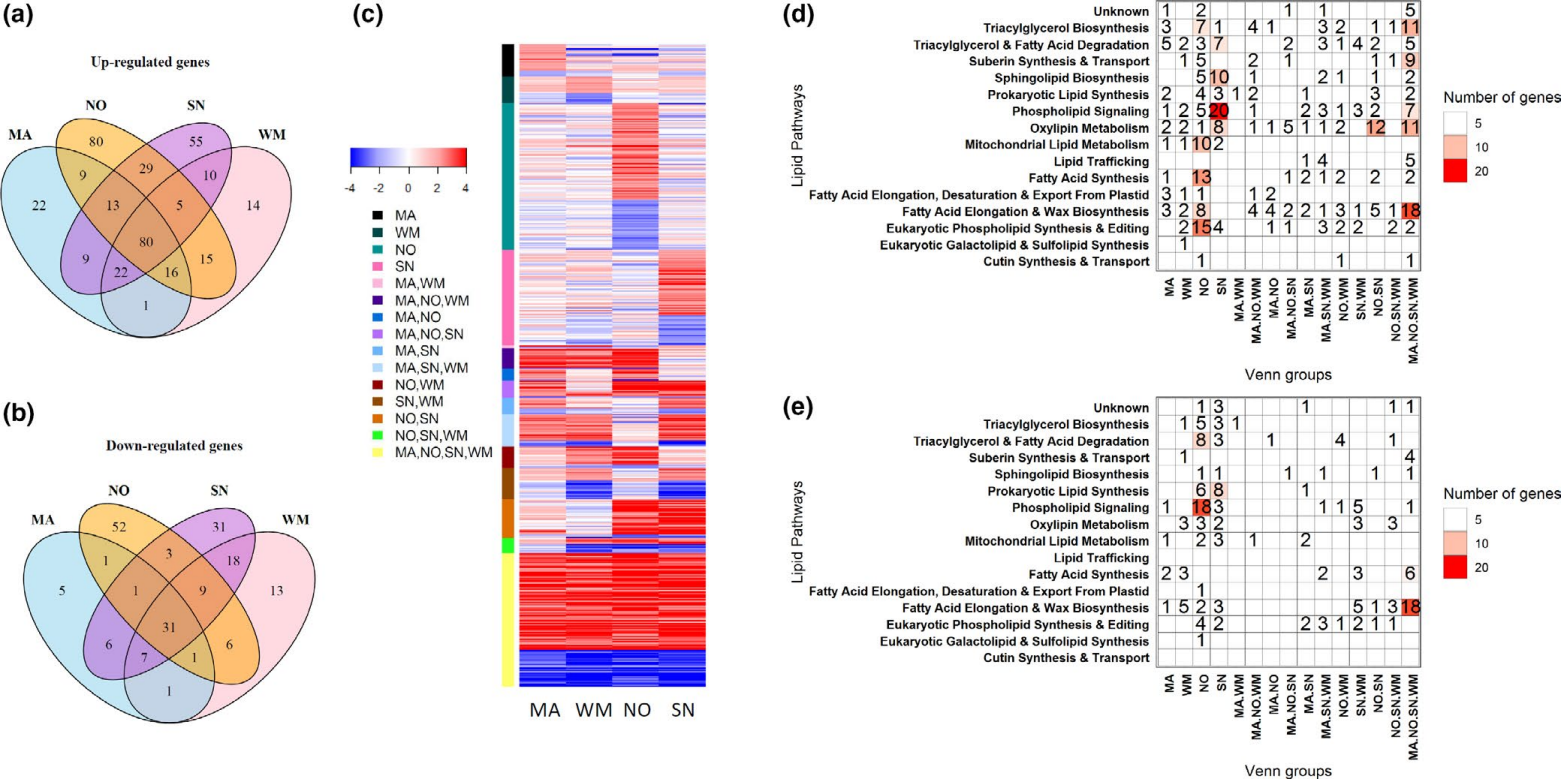
Differentially expressed genes (DEGs) involved in lipid metabolism. DEGs in lipid metabolism were identified in MA, WM, SN, and NO between cold treatment (4°C) and control (22°C), respectively. Venn diagrams for upregulated (a) or downregulated (b) genes in lipid metabolism were generated. Heatmap (c) displays log_2_ fold change (log2FC) values of DEGs categorized from Venn groups. (d) and (e) display the number of genes in the glycerolipid pathways. MA, Manitou; WM, winter Manitou; NO, Norstar; SN, Spring Norstar

We specifically examined genes in lipid metabolism. A total of 1,268 lipid genes were identified based on orthologous lipid genes in *A*. *thaliana*. Of these lipid genes, 225, 248, 350, and 328 genes were significantly changed in MA, WM, NO, and SN after 6‐week cold treatment (4°C), compared with their respective controls at 22°C. We further categorized the DEGs into different groups using Venn diagrams (Figure 4). Specifically, 10, 13, and 15 genes in mitochondrial lipid metabolism, fatty acid synthesis, and eukaryotic phospholipid synthesis and editing pathway were upregulated in NO (Figure 4d); 20 genes from phospholipid signaling pathway was upregulated in SN, whereas 18 genes were downregulated in NO (Figure 4e). Overall, more DEGs were altered in NO and SN lines, suggesting an active basal response in wheat lines with NO genetic background.

**FIGURE 5:**
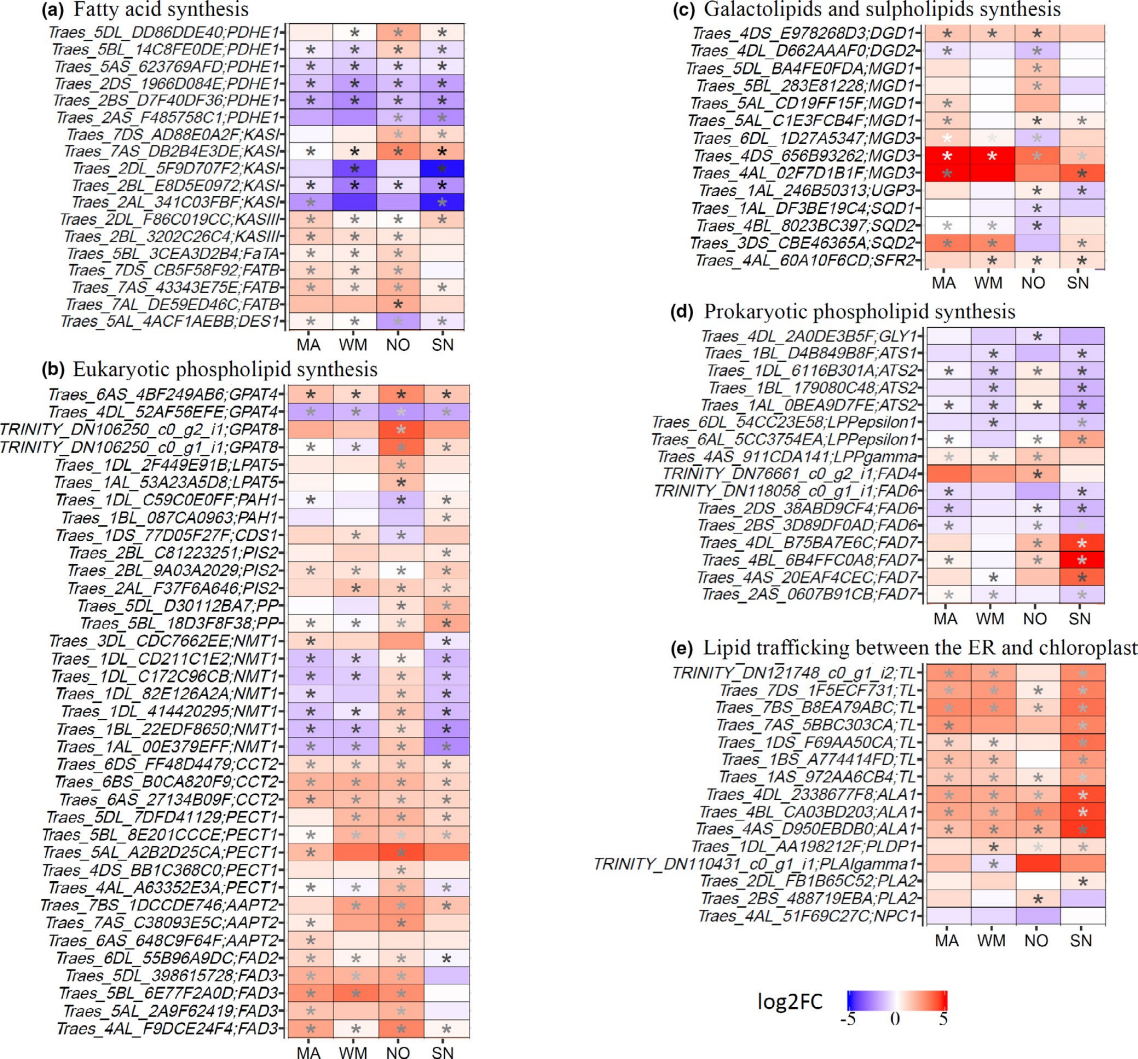
Differentially expressed genes (DEGs) in glycerolipid pathways. DEGs were identified in MA, WM, SN, and NO between cold treatment (4°C) and control (22°C), respectively. Glycerolipid pathways were generally classified into (a) fatty acid biosynthesis, (b) eukaryotic phospholipid synthesis, (c) galactolipids and sulpholipids synthesis, (d) prokaryotic phospholipid synthesis, and (e) lipid trafficking between the endoplasmic reticulam and chloroplast. Heatmaps display log_2_ fold change (log2FC) values for each comparison and statistical significance between cold treatment (4°C) to control (22°C) are shown as asterisk (*p‐value < .01). Rows represent genes as indicated by specific gene names on the y axis. Columns represent comparisons between cold treatment (4°C) and control (22°C). MA, Manitou; WM, winter Manitou; NO, Norstar; SN, Spring Norstar

We further grouped lipid genes into different glycerolipid biosynthetic pathways (Figure 5). Consistent with the aforementioned qRT‐PCR results, RNA‐seq results showed that genes encoding FAD4, which solely catalyzes the desaturation of 16:0 to t16:1 in PG were upregulated at 4°C in all four wheat varieties (Figure 5d), despite that t16:1 in PG content was decreased during cold acclimation. Our data also revealed that genes encoding pyruvate dehydrogenase complex E subunit (PDHE1), responsible for acetyl‐CoA provision, were decreased, thereby suggesting a reduced fatty acid synthesis. On the other hand, genes encoding fatty acid thioesterase, which participates in releasing fatty acids from acyl‐ACP for channeling into the cytosolic compartment, were induced. Significantly, genes concerning eukaryotic phospholipid synthesis and lipid editing in the cytosolic compartment, such as fatty acid desaturase 2 (*FAD2*), fatty acid desaturase 3 (*FAD3*), phosphorylethanolaminecytidylyltransferase1 (*PECT1*), were mostly induced. Major genes involved in the prokaryotic glycerolipid pathway, including the plastid‐localized *glycerol*‐*3*‐*phosphate dehydrogenase* (*GLY1*), *glycerol*‐*3*‐*phosphate acyltransferase* (*ATS1*), *lysophosphatidic acid acyltransferase 1* (*LPAT1* or *ATS2*), and *fatty acid desaturase 6* (*FAD6*) were downregulated. Furthermore, several genes involved in lipid trafficking pathway, such as *translocase* (*TL*s) and *aminophospholipid ATPase* (*ALA*s), were upregulated, suggesting an enhanced lipid trafficking between the ER to chloroplast. Hence, consistent with our biochemical analysis, transcriptome analysis also illustrated a reduced chloroplast pathway and an increased eukaryotic pathway in leaf tissues under cold treatment.

## 4 DISCUSSION

Freezing tolerance in plants is a critical factor limiting the agricultural range of many crops. Studies have previously reported that changes in the t16:1 content of PG after exposure to cold acclimation temperature displayed a correlation with freezing capacity in cereals (Gray et al., 2005; Huner et al., 1987, 1989; Murata, 1983). Extending from this, we found that the decrease in t16:1 level proceeded at a pace similar to that of cold hardiness development in four selected wheat lines under cold acclimation conditions (Fowler & Limin, 2004). MA and the NIL SN (NO harboring the spring vernalization loci *Vrn*‐*A1*) reached their lowest t16:1 content in 4 weeks, which was also the time they required to achieve full acclimation. NO and the NIL WM (MA harboring the winter vernalization loci *vrn*‐*A1*) took 6 weeks to decline to the lower most level of t16:1, at a time point when their cold harden state was reached. More importantly, we demonstrated that this fatty acid composition changes to be relevant in field conditions, confirming that t16:1 dynamics is a reliable metabolite ideotype for assessing the capacity of freezing tolerance in wheat.

Phosphatidylglycerol unsaturation was previously hypothesized to be consequential to chilling sensitivity (Murata et al., 1992; Roughan, 1985). However, conversion of 16:0 to t16:1 would not have impact as far as modulation of membrane fluidity is concerned because the physical properties of t16:1 resembles that of saturated fatty acid (Roughan, 1985). Recent studies have indicated a coordination between the eukaryotic and prokaryotic pathway leading to alterations in glycerolipid composition on temperature stress (Li et al., 2015). The differences between the two pathways lies in the distinctive DAG moieties derived from the assembly of glycerolipids, which can be of ER origin via the eukaryotic pathway or from plastid through the prokaryotic pathway (Browse et al., 1986; Ohlrogge & Browse, 1995). PG in leaf tissues is primarily synthesized in chloroplast with C16 chain fatty acids present at the *sn*‐2 position, a hallmark for lipids synthesized through the prokaryotic pathway. Our results showed that low temperature treatment led to elevated C18 chain fatty acids at the *sn*‐2 position of PG, indicating an increase in the flow of DAG from the ER to chloroplast for PG synthesis. Moreover, the increase of 16:0, the precursor of t16:1, was found mostly on *sn*‐1, suggesting that the decrease of t16:1 was not due to reduced desaturation activity. Indeed, transcript profile of the *FAD4* gene confirmed that the reduction of t16:1 was unlikely due to reduced desaturation activity. Lipidomics analysis brought forward additional information that supports the above supposition because PG molecular species such as PG34:3 (16:0/18:3) and PG36:6 (18:3/18:3) were all increased after cold treatment. Taken together, we conclude that the decreased t16:1 in leaf tissues after cold acclimation reflected a decreased contribution from the prokaryotic glycerolipid pathway to chloroplast lipid synthesis.

Despite of the apparent correlation, our results do not support a direct causal relationship between the decrease in t16:1 and cold acclimation. Existing literature indicates that molecular events dictating cold hardiness capacity occurs primarily in crown tissues. In harsh climate such as that of the Canadian prairies, leaf tissues do not survive in winter, and it is the crown tissue beneath the ground that ensures the spring growth. Our results showed that the presence of t16:1 in crown tissues is negligible, which is not surprising because crown tissues do not have photosynthetic plastids. Consequently, reduction of t16:1 is not expected to contribute to modulation of membrane fluidity (Murata & Los, 1997). We speculate that the onset of cold hardiness development in the crown tissue and the suppression of the chloroplast glycerolipid pathway in leaves are regulated by a similar signaling cascade that is triggered by low temperature exposure. Such an apparent correlation between a specific fatty acid composition change and development of freezing tolerance traits presents t16:1 as an interesting metabolic ideotype for screening winter hardiness and a valuable tool for dissecting genetic determinants governing the capacity of freezing tolerance.

## CONFLICT OF INTEREST

The authors declare no conflict of interest.

## Supporting information

Supplementary Material

Supplementary Material

Supplementary Material

Supplementary Material

Supplementary Material
