# Supplementary material for: Elucidating the biochemical basis of trans‐16:1 fatty acid change in leaves during cold acclimation in wheat
Source: Plant Environ Interact. 2021 May 17;2(3):101–11. doi: 10.1002/pei3.10044 (PMC10168083; doi:10.1002/pei3.10044)
Supplement: Supplementary file 5 — Supplementary Material [file PEI3-2-101-s003.pdf]

Supplementary Tables and Figure Legends

Supplementary Tables

Supplementary Table 1. Manitou glycerolipid profile of cold-treated and non-treated wheat leaves.

Supplementary Table 2. Winter Manitou glycerolipid profile of cold-treated and non-treated wheat leaves.

Supplementary Table 3. Norstar glycerolipid profile of cold-treated and non-treated wheat leaves.

Supplementary Table 4. Spring Norstar glycerolipid profile of cold-treated and non-treated wheat leaves.

Supplementary Table 5. Primers used in this study.

Supplementary Figures

Supplementary Figure 1. Soil temperature in the field trial in year 2013 and 2014.

Supplementary Figure 2. The percentage decrease in t16:1 fatty acid in cold acclimated wheat lines under different temperatures.

Supplementary Figure 3. The percentage decrease in t16:1 during cold acclimation at 4°C after two, four, and six weeks.

Supplementary Figure 4. Fatty acid profile of phosphatidylglycerol (PG) isolated from crown tissues of cold and non-cold treated (Control) wheat. MA-Manitou, WM-Winter Manitou, SN-Spring Norstar, NO-Norstar.

Supplementary Figure 5. Alignment of partial cDNA sequences of *TaFAD4* with Arabidopsis (*AtFAD4*) and Brachypodium *FAD4* gene.

Supplementary Figure 6. Alignment of predicted amino acid sequence of a putative wheat FAD4 (F9) with its homologs from Arabidopsis (AtFAD4) and Brachypodium.

Supplementary Figure 7. Expression of *TaFAD4* in wheat lines after cold acclimation. (A) RT-PCR results of *TaFAD4*. (B) Average expression level of *TaFAD4* identified in RNA-seq via *de novo* assembly.

Supplementary Figure 8. Lipidomic analysis of phosphatidylglycerol (PG) in crown and leaf tissues in four wheat lines under control (22°C) and cold treatment (4°C).

Supplementary Figure 9. Sample-wise and gene-wise clustering of DEGs and lipid traits.

Supplementary Figure 10. GO enrichment analysis of clusters.

Supplementary Dataset

Supplementary Data 1. Lipidomics analysis for leaf and crown tissues during cold acclimation.

Supplementary Data 2. Detailed statistics of mapping RNA-seq reads to the wheat genome and unmapped reads to *de novo* assembled genes.

Supplementary Data 3. Lists of DEGs in MA, NO, WM and SN.

Supplementary Data 4. Gene ontology (GO) enrichment for the clusters of differentially expressed genes.

Supplementary Tables

Supplementary Table 1. Manitou glycerolipid profile of cold-treated (treatment) and non-treated (control) wheat leaves. DGDG, digalactosyldiacylglycerol; MGDG, monogalactosyldiacylglycerol; PC, phosphatidylcholine; PE, phosphatidylethanolamine; PG, phosphatidylglycerol; PI, phosphatidylinositol; SQDG, sulfoguinovosyldiacylglycerol; TFA, total fatty acids. Statistically significant differences (two-tailed Student's t test) were calculated from three biological replicates (n=3) between treatment (4°C) to control (22°C), respectively. *, P<0.05.

			Fatty acids composition (mol%)						Total lipids
			16:0	t16:1	18:0	18:1c9	18:2	18:3	(%)
Manitou	PI	control	50.4 ± 3.09	nd	1.92 ± 0.25	0.78 ± 0.69	19.9 ± 0.9	26.8 ± 2.5	1.58 ± 0.12
		treatment	49.4 ± 0.46*	0.15 ± 0.25	1.09 ± 0.25*	0.89 ± 0.06	16.8 ± 0.46*	31.5 ± 0.56*	2.56 ± 0.11*
	PC	control	31.1 ± 0.53	0.44 ± 0.03	0.96 ± 0.17	3.96 ± 0.09	29.8 ± 0.21	33.1 ± 0.54	10.5 ± 0.66
		treatment	25.2 ± 0.02*	0.26 ± 0.01*	0.4 ± 0.24*	1.65 ± 0.89*	20.4 ± 1.58	37.9 ± 7.97*	14.9 ± 1.2*
	PE	control	28.6 ± 1.05	0.12 ± 0.21	1.29 ± 0.29	1.31 ± 0.19	45.0 ± 1.06	23.1 ± 0.84	3.97 ± 0.58
		treatment	28.6 ± 0.81	0.25 ± 0.01	0.63 ± 0.24*	0.93 ± 0.1*	37.2 ± 0.31*	32.1 ± 0.91*	8.26 ± 0.95*
	PG	control	13.2 ± 0.77	44.2 ± 0.41	1.01 ± 0.51	1.13 ± 0.09	6.25 ± 0.15	33.8 ± 1.43	10.3 ± 0.61
		treatment	21.2 ± 0.37*	35.8 ± 0.36*	0.7 ± 0.09	1.29 ± 0.03*	7.83 ± 0.17*	32.9 ± 2.85	10.8 ± 2.85
	DGDG	control	10.3 ± 0.16	0.37 ± 0.04	0.81 ± 0.04	0.27 ± 0.02	2.73 ± 0.1	85.7 ± 0.26	22.3 ± 0.35
		treatment	9.27 ± 0.45*	0.26 ± 0.04*	0.59 ± 0.09*	0.29 ± 0.03	1.68 ± 0.22*	87.6 ± 0.82*	24.2 ± 1.8
	MGDG	control	1.6 ± 0.06	0.22 ± 0.02	0.14 ± 0.02	0.23 ± 0.01	4.35 ± 0.04	93.4 ± 0.12	45.3 ± 1.64
		treatment	3.03 ± 0.22*	0.19 ± 0.04	0.26 ± 0.02*	0.29 ± 0.07	2.37 ± 0.17*	93.7 ± 0.21*	33.1 ± 1.91*
	SQDG	control	30.3 ± 1.23	0.39 ± 0.36	1.35 ± 0.23	0.27 ± 0.01	4.59 ± 0.04	63.0 ± 1.77	5.76 ± 0.27
		treatment	28.6 ± 3.28*	0.33 ± 0.28	1.1 ± 0.15	0.05 ± 0.09*	1.09 ± 0.07*	68.7 ± 3.58*	5.61 ± 1.07*
	TFA	control	12.4 ± 0.25	4.6 ± 0.07	1.06 ± 0.7	0.88 ± 0.01	9.63 ± 0.16	71.0 ± 0.82	
		treatment	16.0 ± 0.44*	3.2 ± 0.22*	0.87 ± 0.14	1.04 ± 0.18	12.9 ± 0.23*	65.6 ± 0.7*	

Supplementary Table 2. Winter Manitou glycerolipid profile of cold-treated (treatment) and non-treated (control) wheat leaves. DGDG, digalactosyldiacylglycerol; MGDG, monogalactosyldiacylglycerol; PC, phosphatidylcholine; PE, phosphatidylethanolamine; PG, phosphatidylglycerol; PI, phosphatidylinositol; SQDG, sulfoguinovosyldiacylglycerol; TFA, total fatty acids. Statistically significant differences (two-tailed Student’s t test) were calculated from three biological replicates (n=3) between treatment (4°C) to control (22°C), respectively. *, P<0.05.

			Fatty acids composition (mol%)						Total lipids
			16:0	t16:1	18:0	18:1c9	18:2	18:3	(%)
Winter Manitou	PI	control	52.0 ± 2.95	nd	3.37 ± 1.39	0.76 ± 0.66	19.3 ± 0.36	24.4 ± 4.43	1.76 ± 0.11
		treatment	50.6 ± 0.94*	0.34 ± 0.6	1.08 ± 0.17*	0.64 ± 0.6	16.8 ± 0.15*	30.4 ± 1.26*	2.15 ± 0.15*
	PC	control	30.2 ± 0.75	0.41 ± 0.05	0.99 ± 0.26	4.39 ± 0.24	32.1 ± 0.36	31.4 ± 0.57	11.0 ± 0.78
		treatment	23.4 ± 0.31*	0.23 ± 0.01*	0.44 ± 0.04*	2.54 ± 0.17*	28.7 ± 0.69*	44.6 ± 0.9*	16.5 ± 0.52*
	PE	control	29.5 ± 1.44	0.13 ± 0.23	0.96 ± 0.3	1.34 ± 0.11	45.6 ± 0.11	22.0 ± 1.36	4.46 ± 0.26
		treatment	26.5 ± 0.79*	0.26 ± 0.07	0.33 ± 0.1*	0.98 ± 0.11*	37.1 ± 0.25*	34.5 ± 1.11*	10.4 ± 0.76*
	PG	control	13.7 ± 0.54	42.2 ± 0.78	0.77 ± 0.18	1.2 ± 0.04	6.72 ± 0.13	34.1 ± 2.18	10.6 ± 0.69
		treatment	21.0 ± 0.79*	30.7 ± 0.57*	0.44 ± 0.06*	0.84 ± 0.08*	7.67 ± 0.24*	39.1 ± 1.20*	10.1 ± 0.65
	DGDG	control	10.5 ± 0.19	0.46 ± 0.08	0.82 ± 0.06	0.3 ± 0.01	2.7 ± 0.02	84.8 ± 0.18	22.5 ± 1.27
		treatment	9.01 ± 0.7*	0.35 ± 0.07*	0.61 ± 0.12*	0.31 ± 0.1	1.53 ± 0.22*	87.8 ± 1.19*	22.6 ± 2.35
	MGDG	control	1.63 ± 0.08	0.22 ± 0.04	0.14 ± 0.02	0.24 ± 0.01	4.21 ± 0.07	93.4 ± 0.1	44.7 ± 0.99
		treatment	2.49 ± 0.15*	0.15 ± 0.01*	0.18 ± 0.03	0.29 ± 0.06	2.28 ± 0.26*	94.5 ± 0.47*	33.7 ± 0.72*
	SQDG	control	29.7 ± 0.54	0.37 ± 0.33	1.68 ± 0.35	0.29 ± 0.05	4.35 ± 0.07	63.5 ± 0.42	4.75 ± 0.51
		treatment	27.8 ± 0.42*	0.26 ± 0.23*	1.04 0.09	0.13 ± 0.11	1.17 ± 0.29*	69.4 ± 0.91	4.69 ± 0.64
	TFA	control	12.9 ± 0.73	4.57 ± 0.16	1.32 ± 0.87	0.98 ± 0.01	10.4 ± 0.16	69.5 ± 1.74	
		treatment	14.9 ± 0.26*	2.78 ± 0.05*	0.78 ± 0.29	0.77 ± 0.45	12.6 ± 0.01*	67.7 ± 0.08	

Supplementary Table 3. Norstar glycerolipid profile of cold-treated (treatment) and non-treated (control) wheat leaves. DGDG, digalactosyldiacylglycerol; MGDG, monogalactosyldiacylglycerol; PC, phosphatidylcholine; PE, phosphatidylethanolamine; PG, phosphatidylglycerol; PI, phosphatidylinositol; SQDG, sulfoguinovosyldiacylglycerol; TFA, total fatty acids. Statistically significant differences (two-tailed Student’s t test) were calculated from three biological replicates (n=3) between treatment (4°C) to control (22°C), respectively. *, P<0.05.

			Fatty acids composition (mol%)						Total lipids
			16:0	t16:1	18:0	18:1c9	18:2	18:3	(%)
Norstar	PI	control	45.3 ± 8.9	1.07 ± 1.86	2.17 ± 0.44	1.58 ± 0.31	18.4 ± 2.49	31.3 ± 9.72	1.63 ± 0.24
		treatment	50.8 ± 1.03	0.21 ± 0.1	0.68 ± 0.59*	0.52 ± 0.46*	17.4 ± 0.67	30.6 ± 0.43	2.57 ± 0.24*
	PC	control	31.3 ± 1.18	0.41 ± 0.02	1.25 ± 0.44	4.75 ± 0.62	29.8 ± 3.33	32.9 ± 4.56	10.7 ± 0.12
		treatment	25.8 ± 0.46*	0.23 ± 0.01*	0.39 ± 0.03*	2.07 ± 0.2*	29.0 ± 0.37	42.3 ± 0.52	16.3 ± 0.93*
	PE	control	31.6 ± 0.28	0.12 ± 0.2	1.85 ± 0.09	1.51 ± 0.04	43.6 ± 0.62	21.2 ± 0.78	3.98 ± 0.44
		treatment	27.5 ± 0.69*	0.26 ± 0.06	0.39 ± 0.03*	0.81 ± 0.08*	37.4 ± 0.42*	33.3 ± 0.34*	10.3 ± 0.77*
	PG	control	12.6 ± 0.15	43.7 ± 0.35	0.73 ± 0.19	1.35 ± 0.02	6.46 ± 0.26	34.7 ± 0.18	11.7 ± 0.81
		treatment	26.0 ± 0.49*	23.4 ±1.06*	0.65 ± 0.19	0.82 ± 0.06*	8.23 ± 0.22*	40.7 ± 1.18*	8.96 ± 0.41*
	DGDG	control	12.1 ± 0.33	0.55 ± 0.15	0.88 ± 0.12	0.43 ± 0.03	3.2 ± 0.08	82.4 ± 0.36	20.7 ± 2.41
		treatment	9.7± 0.47*	0.35 ± 0.02*	0.48 ± 0.01*	0.32 ± 0.03*	1.65 ± 0.13*	87.2 ± 0.63*	24.7 ± 0.09*
	MGDG	control	1.84 ± 0.06	0.16 ± 0.03	0.17 ± 0.02	0.32 ± 0.01	4.73 ± 0.06	92.6 ± 0.13	45.8 ± 2.83
		treatment	2.25 ± 0.15*	0.04 ± 0.04	0.17 ± 0.03	0.24 ± 0.06*	2.1 ± 0.14*	94.9 ± 0.35*	32.6 ± 1.85*
	SQDG	control	32.3 ± 1.04	0.57 ± 0.12	1.28 ± 0.29	0.51 ± 0.26	4.22 ± 0.05	60.5 ± 1.86	5.33 ± 0.44
		treatment	34.1 ± 3.2*	0.52 ± 0.03	1.13 ± 0.14	0.1 ± 0.09*	0.97 ± 0.19*	63.0 ± 3.01	4.42 ± 0.89*
	TFA	control	13.8 ± 1.35	4.85 ± 0.14	1.31 ± 1.05	1.14 ± 0.04	10.0 ± 0.39	68.4 ± 1.84	
		treatment	16.2 ± 0.27*	2.53 ± 0.15*	0.58 ± 0.02	0.88 ± 0.12*	13.1 ± 0.22*	66.3 ± 0.77	

Supplementary Table 4. Spring Norstar glycerolipid profile of cold-treated (treatment) and non-treated (control) wheat leaves. DGDG, digalactosyldiacylglycerol; MGDG, monogalactosyldiacylglycerol; PC, phosphatidylcholine; PE, phosphatidylethanolamine; PG, phosphatidylglycerol; PI, phosphatidylinositol; SQDG, sulfoguinovosyldiacylglycerol; TFA, total fatty acids. Statistically significant differences (two-tailed Student's t test) were calculated from three biological replicates (n=3) between treatment (4°C) to control (22°C), respectively. *, P<0.05.

			Fatty acids composition (mol%)						Total lipids
			16:0	t16:1	18:0	18:1c9	18:2	18:3	(%)
Spring Norstar	PI	control	50.5 ± 1.08	0.42 ± 0.2	2.62 ± 1.4	1.33 ± 0.23	16.3 ± 2.67	28.7 ± 1.26	1.79 ± 0.61
		treatment	49.8 ± 1.31	0.4 ± 0.3	1.29 ± 0.17	0.99 ± 0.05*	17.8 ± 0.44	30.5 ± 1.78	2.3 ± 0.16
	PC	control	31.5 ± 0.64	0.42 ± 0.07	0.99 ± 0.27	5.2 ± 0.07	26.5 ± 0.28	35.0 ± 0.72	11.0 ± 0.19
		treatment	25.6 ± 0.36*	0.25 ± 0.01*	0.52 ± 0.02*	2.8 ± 0.13*	30.3 ± 0.23*	40.4 ± 0.22*	16.3 ± 0.24*
	PE	control	30.6 ± 0.73	nd	0.98 ± 0.28	1.56 ± 0.02	42.0 ± 0.51	24.8 ± 0.67	3.69 ± 0.66
		treatment	27.9 ± 0.63*	0.27 ± 0.07*	0.58 ± 0.06*	0.96 ± 0.07*	38.0 ± 0.51*	32.0 ± 0.52*	9.58 ± 1.94*
	PG	control	13.5 ± 0.53	44.0 ± 0.84	0.72 ± 0.13	1.37 ± 0.01	6.4 ± 0.03	33.6 ± 1.4	11.1 ± 0.66
		treatment	22.4 ± 0.94*	31.5 ± 0.73*	0.64 ± 0.17	0.84 ± 0.04*	7.3 ± 0.15*	37.1 ± 1.45*	7.67 ± 2.14*
	DGDG	control	11.9 ± 0.73	0.36 ± 0.11	0.74 ± 0.01	0.46 ± 0.01	3.52 ± 0.06	82.6 ± 0.82	20.6 ± 0.7
		treatment	9.6 ± 0.41*	0.29 ± 0.02	0.52 ± 0.01*	0.37 ± 0.04*	1.79 ± 0.13*	87.1 ± 0.6*	25.3 ± 1.26*
	MGDG	control	1.97 ± 0.33	0.22 ± 0.03	0.16 ± 0.03	0.37 ± 0.02	3.77 ± 0.79	93.4 ± 2.38	46.7 ± 2.1
		treatment	2.58 ± 0.27*	0.25 ± 0.06	0.19 ± 0.04	0.27 ± 0.04*	2.19 ± 0.05	94.4 ± 0.37	34.1 ± 1.65*
	SQDG	control	32.3 ± 0.26	0.48 ± 0.03	1.21 ± 0.04	0.35 ± 0.02	4.31 ± 0.07	61.2 ± 0.09	5.01 ± 0.6
		treatment	32.8 ± 1.44	0.55 ± 0.04*	1.11 ± 0.02*	0.5 ± 0.08*	0.86 ± 0.12*	64.5 ± 1.19*	4.56 ± 0.53
	TFA	control	13.5 ± 0.79	4.79 ± 0.11	0.95 ± 0.57	1.17 ± 0.02	9.9 ± 0.2	69.3 ± 1.03	
		treatment	15.4 ± 0.87*	2.86 ± 0.07*	0.58 ± 0.05	1.03 ± 0.17	12.8 ± 0.47*	66.9 ± 1.69	

Supplementary Table 5. Primers used in this study.

Name	Sequence(5' to 3')	Comments
JW560	ACGAACTGTCAGTAACAGCGACGGCGG	for 5'RACE
JW561	GGAAGAAGACGACCATCTCCAGCGCCT	for 5'RACE
JW560	ACGAACTGTCAGTAACAGCGACGGCGG	for RT-PCR
JW566	TACGTGGAAGCAGACACAAGC	for RT-PCR
JW525	TGTCGCACCAGAGGATCATC	actin
JW526	TTCCAGCTCCTGCTCATAATCA	actin

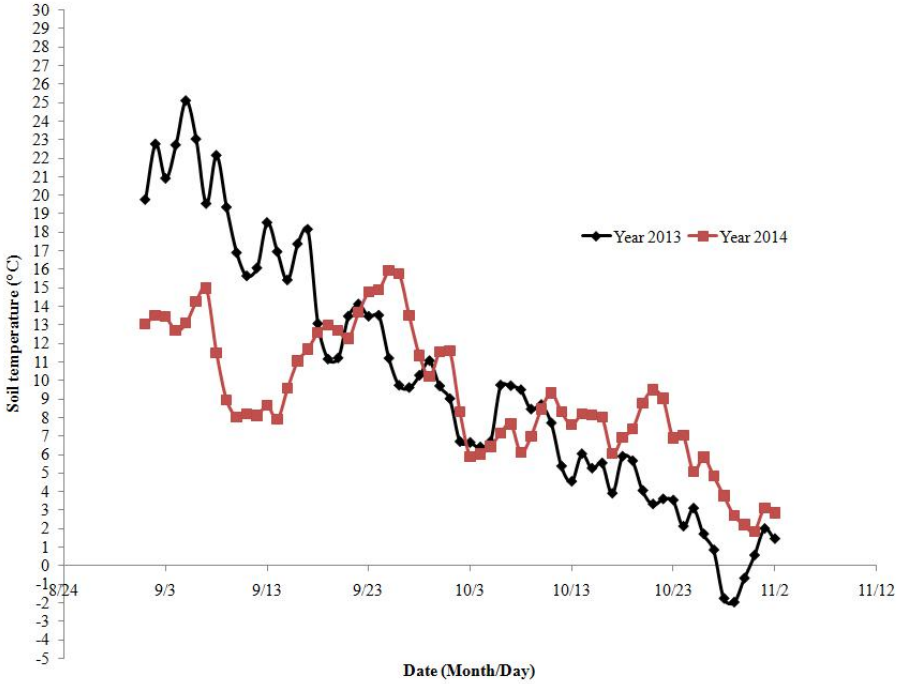

Supplementary Figure 1. Soil temperature in the field trial site in year 2013 and 2014. Seeds was sown at the end of August. Samples were harvested on September 23 (13.4°C) and October 23(3.5°C) in the 2013 trial, and September 22(13.7°C) and October 30 (2.2°C) in the 2014 trial.

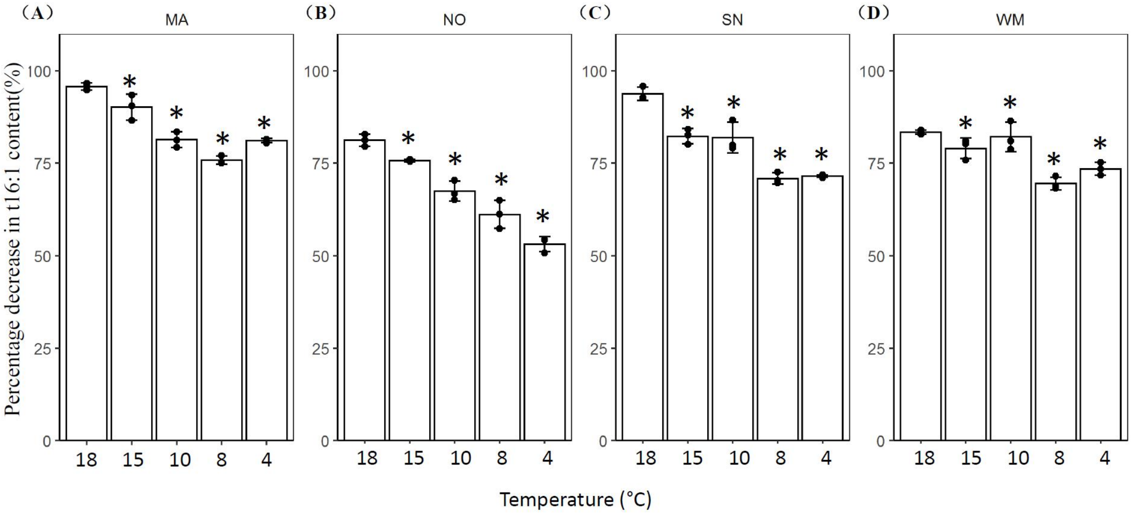

Supplementary Figure 2. The percentage decrease in t16:1 fatty acid in cold acclimated wheat lines under different temperatures. Plants were grown at 22 °C till three-leaf-stage (control) and then transferred to tested temperatures (18°C, 15°C, 10°C, 8°C and 4°C) for an additional six-week treatment. Low temperature-induced changes in t16:1 were calculated based on percentages of decrease between cold treated and control samples. Values are expressed as means ± SD (n=3). Statistically significant differences (two-tailed Student's *t* test) were calculated between cold treatments and control (22 ° C), respectively. *, $P<0.05$.

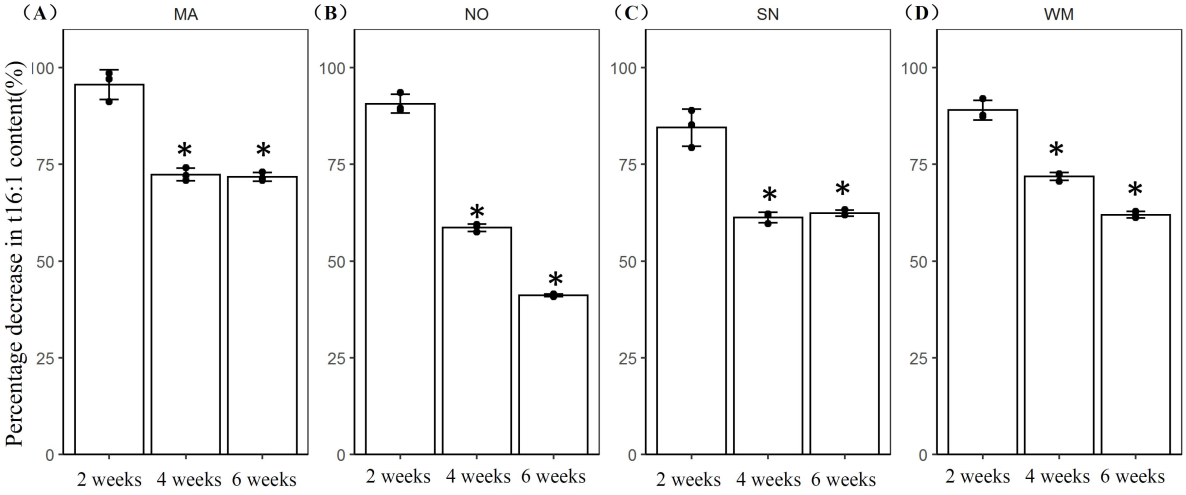

Supplementary Figure 3. The percentage decrease in t16:1 during cold acclimation at 4°C after two, four, and six weeks. Plants were grown at 22 °C till three-leaf-stage and then transferred to 4°C for two, four and six weeks of cold treatment. The percentage decrease in t16:1 after cold treatment (4°C) was calculated relative to that of the t16:1 content in control (22 °C). Statistically significant differences (two-tailed Student's *t* test) were calculated from three biological replicates (n=3) between cold treatment (4°C) to control (22°C), respectively. *, $P<0.05$.

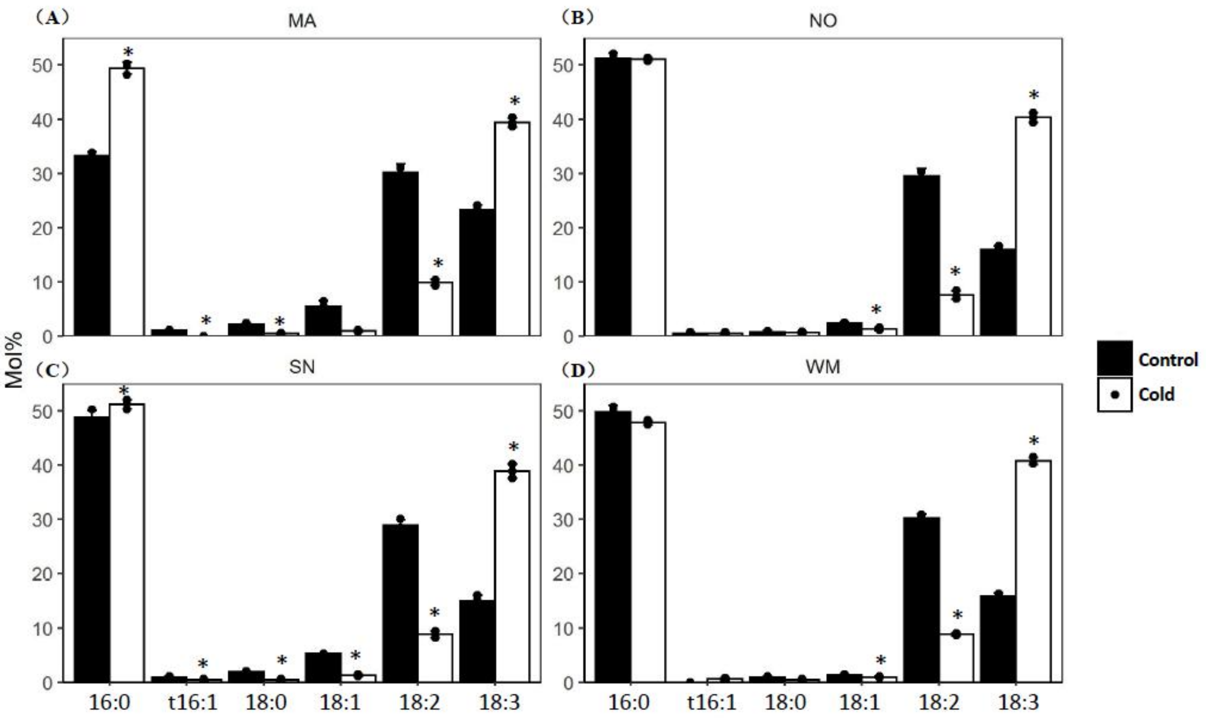

Supplementary Figure 4. Fatty acid profile of phosphatidylglycerol (PG) isolated from crown tissues of cold and non-cold treated (Control) wheat. MA-Manitou, WM-Winter Manitou, SN-Spring Norstar, NO-Norstar. Values are means±SD (n=3). Statistically significant differences (two-tailed Student's *t* test) were calculated from three biological replicates (n=3) between cold treatment (4°C) to control (22°C), respectively. *, $P<0.05$.

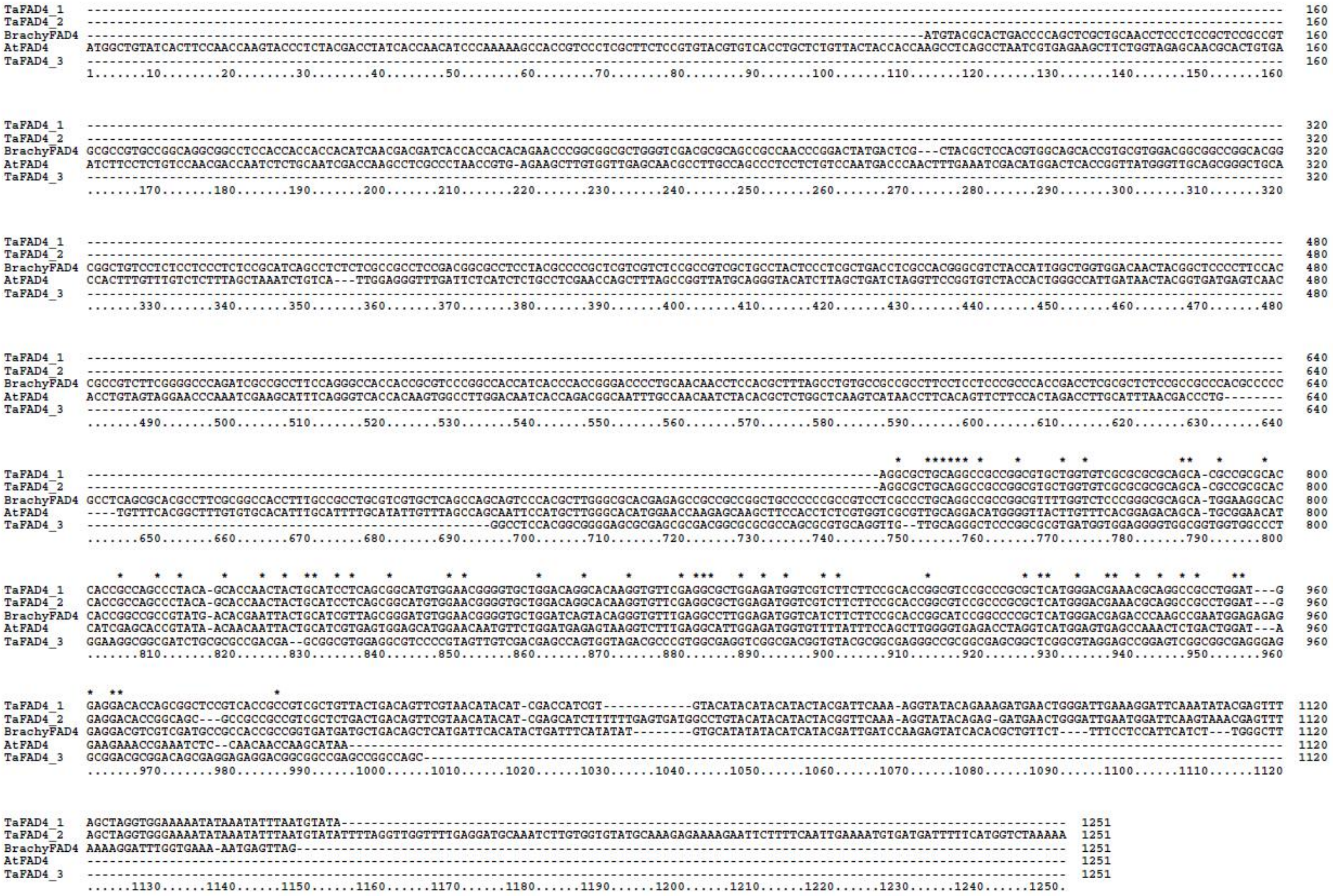

Supplementary Figure 5. Alignment of partial cDNA sequences of *TaFAD4-1, TaFAD4-2, TaFAD4-3* with Arabidopsis (*AtFAD4*) and Brachypodium *FAD4.*

```
AtFAD4              MAVSLPTKYPLRPIT-NIPKSHRPSLLRVRVTCSVTTTKPQPNREKLLVEQRTVNLPLSN        59
Brachypodium        -------MYALTPARCNLPPLRRA-----------------PCRQAASTTTTSTT-----        31
F9 (wheat)          ----MPAMYALTP-RCTLPPVHRRA--------------PPPCRAASPT-----------        30
                            * * *   .:*  :*                  * *    .
ATFAD4              DQSLQSTKPRPNREKLVVEQRLASPPLSNDPTLKSTWTHRLWVAAGCTTLFVSLAKSVIG        119
Brachypodium        ---IT-TTQNP----AALGRR--AAANPDYDSLRSTWQHRAWTAAGTAAVLSSLSASASL        81
F9(wheat)           ----------P----A-------ALARADPDELRSTWPQRAWMLAGSAAVLSSLSASASL        69
                              *            :    :   *:*** :* *  ** :::: **: *.
ATFAD4              GFD-SHLCLEPALAGYAGYILADLGSGVYHWAIDNYGDESTPVVGTQIEAFQGHHKWPWT        178
Brachypodium        AASDGASYAPLVVSAVAAYSLADLATGVYHWLVDNYGSPSTAVFGAQIAAFQGHHRVPAT        141
F9(wheat)           AADSG-SYAEPLAAALAAYTVADLATGVYHWLVDNYGDASTPLVGAQIAAFQGHHRHPST        128
                    . . .        :. *.* :***.:***** :****. ** :.*:** ******: * *
ATFAD4              ITRRQFANNLHALAQVITFTVLPLDLAFN----DPVFHGFVCTFAFCILFSQQFHAWAHG        234
Brachypodium        ITHRDPCNNLHALACAAAFLLPPTDLALSAAHAPASAHAFAATFAACVVLSQQSHAWAHE        201
F9(wheat)           ITRREPCNNLHALARAVALALPAVEGAAAAAHAPAAAHAFAGTFAACVVLSQQFHAWAHE        188
                    **:*: .******* . :: :   : *          *.*. *** *:::*** *****
ATFAD4              TKSKLPPLVVALQDMGLLVSRRQHAEHHRAPYNNNYCIVSGAWNNVLDESKVFEALEMVF        294
Brachypodium        SRRRLPPAVLALQAAGVLVSRAQHGRHHRPPYDTNYCIVSGMWNGVLDQYRVFEALEMVI        261
F9(wheat)           KRRRLPPGIEALQAAGVLVSRAQHAAHHRQPYSTNYCILSGMWNGVLDRHKVFEALEMVV        248
                    .: :*** : ***  *:**** **. *** **..****:** ** ***. :********.
ATFAD4              YFQLGVRPRSWSEPNSDWIEETEISNNQA-------    323
Brachypodium        FFRTGIRPRSWDETQAEWREEDVVDAATAGDDADSS    297
F9(wheat)           FFRTGVRPRSWDETQAAWMEDTSGSVTAVAV-TDSS    283
```

Supplementary Figure 6. Alignment of predicted amino acid sequence of a putative wheat FAD4 (F9) with its homologs from Arabidopsis (AtFAD4) and Brachypodium.

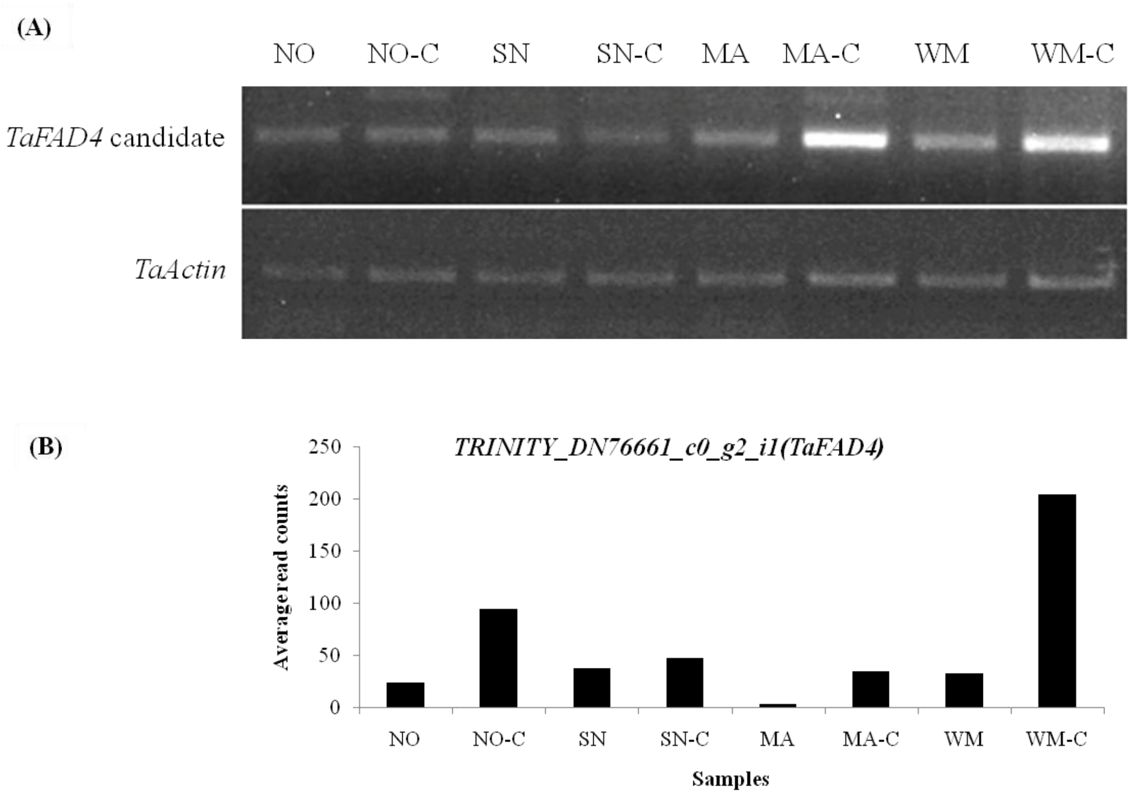

Supplementary Figure 7. Expression of *TaFAD4* in wheat lines after cold acclimation. (A) RT-PCR results of *TaFAD4*. (B) Average of normalized read counts of *TaFAD4* identified in RNA-seq via *de novo* assembly.

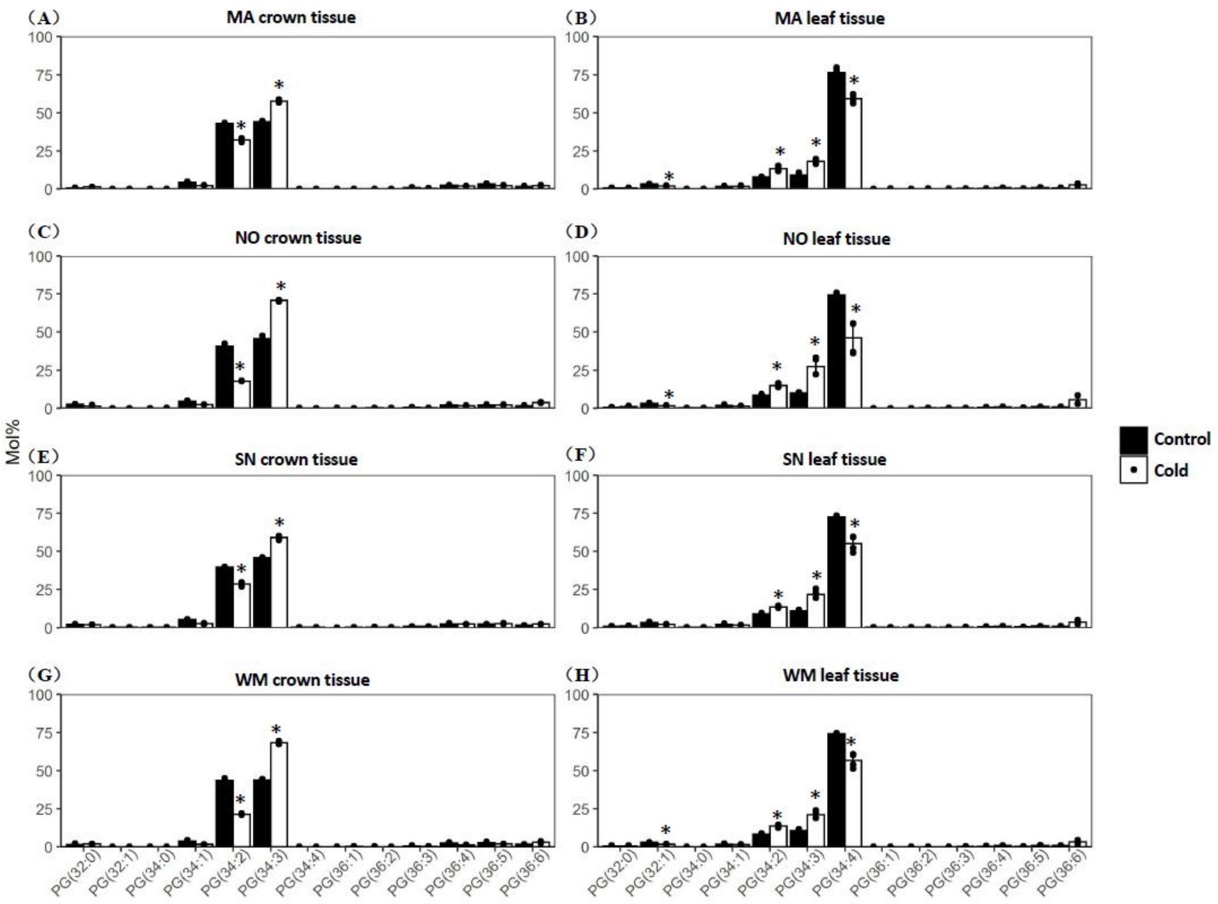

Supplementary Figure 8. Lipidomics analysis of phosphatidylglycerol (PG) in crown and leaf tissue in four wheat lines in the control (22°C) and under cold treatment (4°C). Lipid species in crowns are shown in A, C, E and G. Lipid species in leave are shown in B, D, F and H. MA-Manitou, WM-winter Manitou, SN-spring Norstar, NO-Norstar. Statistically significant differences (two-tailed Student's *t* test) were calculated from four biological replicates (n=4) between cold treatment (4°C) to control (22°C), respectively. *, P<0.05.

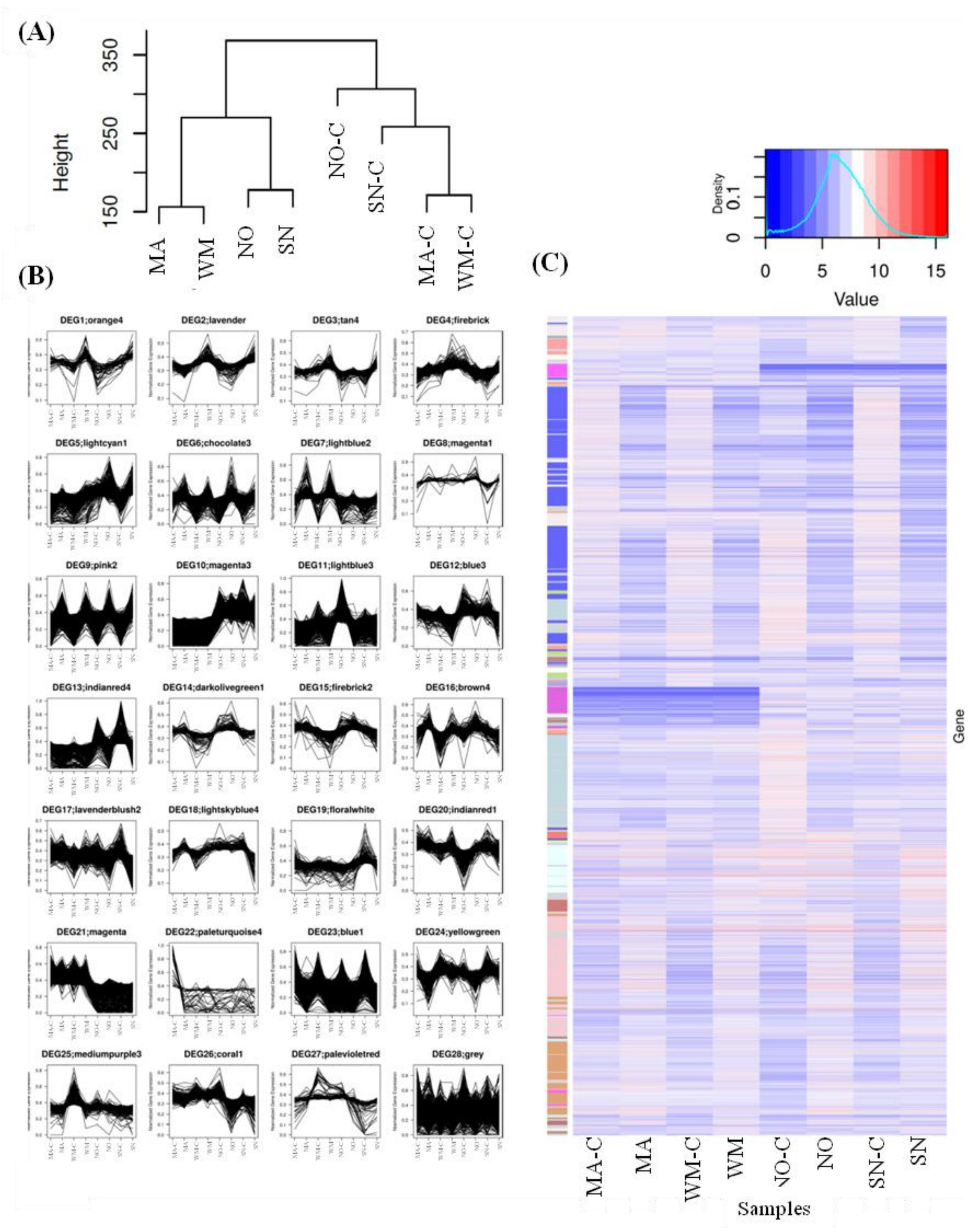

Supplementary Figure 9. Sample-wise and gene-wise clustering of DEGs and lipid traits. A total of 29,491 genes and 233 lipid traits were used for clustering. (A) sample-wise clustering. (B) gene clusters corresponding to the gene-wise clustering. Clusters are named using their sorted numerical order, and colors as in the heatmap (C). (C) heatmap of gene-wise clustering. MA-C: Manitou, Cold Treated; MA: Manitou, Control; WM-C: Winter Manitou, Cold Treated; WM: Winter Manitou, Control; NO-C: Norstar, Cold Treated; NO: Norstar, Control; SN-C: Spring Norstar, Cold Treated; SN: Spring Norstar, Control.

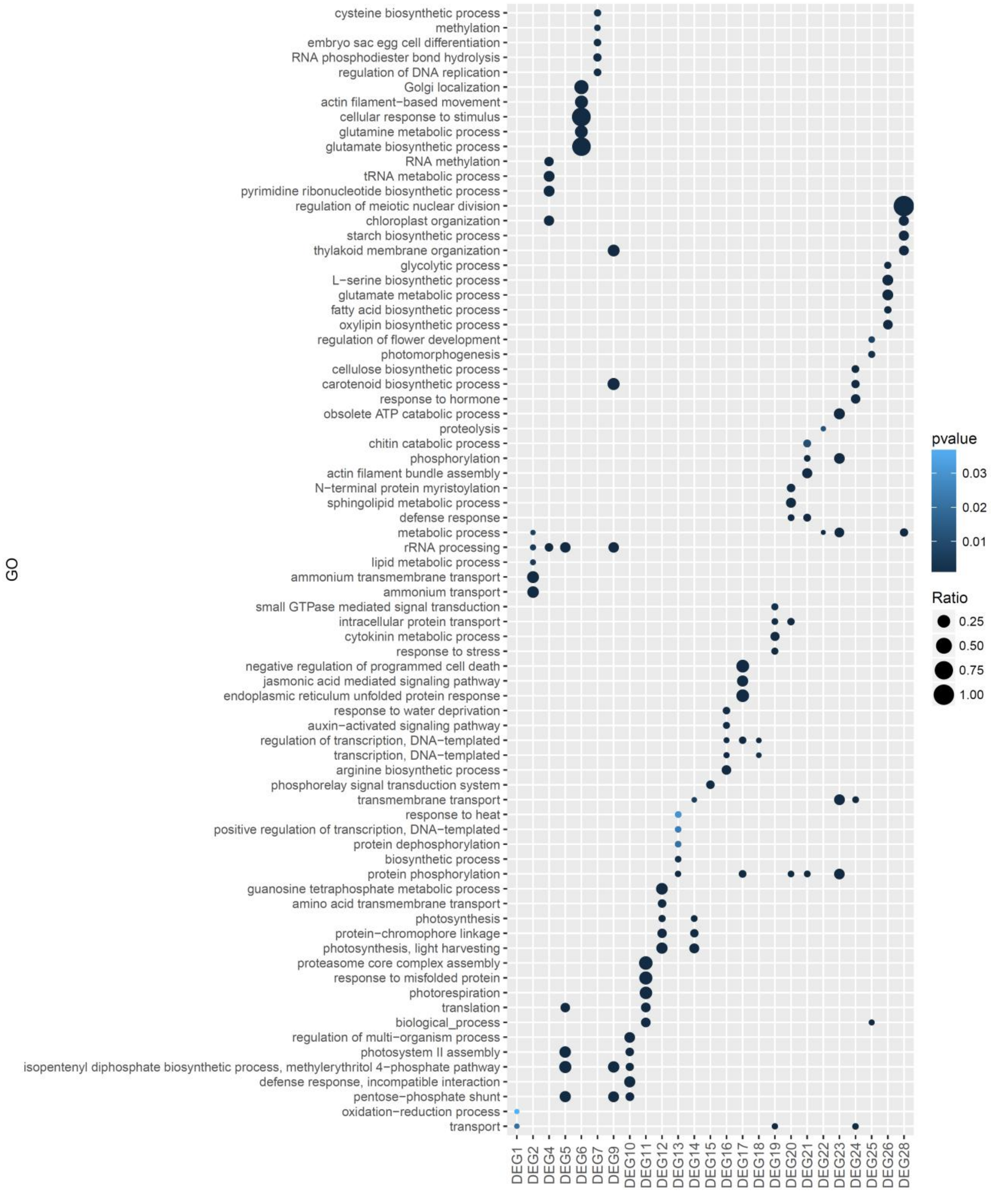

Supplementary Figure 10. Gene ontology (GO) enrichment for the clusters of differentially expressed genes.
